# The absence of specific yeast heat-shock proteins leads to abnormal aggregation and compromised autophagic clearance of mutant Huntingtin proteins

**DOI:** 10.1371/journal.pone.0191490

**Published:** 2018-01-18

**Authors:** Ryan Higgins, Marie-Helene Kabbaj, Alexa Hatcher, Yanchang Wang

**Affiliations:** 1 Department of Biomedical Sciences, College of Medicine, Florida State University, Tallahassee, Florida, United States of America; 2 College of Nursing, Florida State University, Tallahassee, Florida, United States of America; University of Pittsburgh, UNITED STATES

## Abstract

The functionality of a protein depends on its correct folding, but newly synthesized proteins are susceptible to aberrant folding and aggregation. Heat shock proteins (HSPs) function as molecular chaperones that aid in protein folding and the degradation of misfolded proteins. Trinucleotide (CAG) repeat expansion in the Huntingtin gene (*HTT*) results in the expression of misfolded Huntingtin protein (Htt), which contributes to the development of Huntington’s disease. We previously found that the degradation of mutated Htt with polyQ expansion (Htt103QP) depends on both ubiquitin proteasome system and autophagy. However, the role of heat shock proteins in the clearance of mutated Htt remains poorly understood. Here, we report that cytosolic Hsp70 (Ssa family), its nucleotide exchange factors (Sse1 and Fes1), and a Hsp40 co-chaperone (Ydj1) are required for inclusion body formation of Htt103QP proteins and their clearance via autophagy. Extended induction of Htt103QP-GFP leads to the formation of a single inclusion body in wild-type yeast cells, but mutant cells lacking these HSPs exhibit increased number of Htt103QP aggregates. Most notably, we detected more aggregated forms of Htt103QP in *sse1*Δ mutant cells using an agarose gel assay. Increased protein aggregates are also observed in these HSP mutants even in the absence Htt103QP overexpression. Importantly, these HSPs are required for autophagy-mediated Htt103QP clearance, but are less critical for proteasome-dependent degradation. These findings suggest a chaperone network that facilitates inclusion body formation of misfolded proteins and the subsequent autophagic clearance.

## Introduction

Misfolded proteins are prone to aggregate and protein aggregates can cause various deleterious effects within cells. Protein aggregation is linked to several neurodegenerative disorders, including Huntington, Alzheimer, Parkinson and prion diseases [1, 2]. Protein aggregation also contributes to the development of diabetes and cancer [3, 4]. Cells have evolved protein quality control mechanisms to combat the potential toxic effects of aggregated proteins. First, misfolded proteins can be refolded into functional proteins. Second, misfolded proteins can be ubiquitinated and targeted for degradation by proteasomes. In addition, aggregated proteins can be cleared through the autophagy pathway. Often the first line of defense against the generation of unfolded/misfolded proteins depends on heat shock proteins (HSPs), which are abundant molecular chaperones involved in protein folding, refolding, disaggregation and degradation [5]. Thus, it is of interest to understand the roles of chaperones in the response to the expression of aggregation-prone proteins.

Hsp70 chaperones are highly abundant ATPases that bind to hydrophobic regions of substrates to facilitate protein folding [6]. When Hsp70 is bound to ATP, the substrate binding affinity is low, but upon ATP catalysis, ADP-bound Hsp70 shows a high substrate binding affinity [7]. Nucleotide-exchange factors (NEFs) are necessary for the exchange of ADP with ATP, and yeast cells have three distinct types of NEFs, including Hsp110 (Sse1 and Sse2), HspBP1 (Fes1), and a bag domain-containing protein (Snl1). Sse1 is the most abundant Hsp70 NEF, followed by Fes1 at approximately one-fifth the level of Sse1, whereas very low levels of Sse2 and Snl1 are detected [8]. In accordance with their expression levels, most of the Hsp70 NEF functions are associated with Sse1 and Fes1 [9]. Another layer of the chaperone network is the Hsp40 co-chaperones, which enhance the intrinsically low ATPase activity of Hsp70s [10]. Hsp40 co-chaperones also contribute to the substrate specificity of Hsp70s [11]. Therefore, Hsp40s, Hsp70s and NEFs comprise an ATP-dependent chaperone network that maintains proteostasis by facilitating the refolding or degradation of misfolded proteins.

Although the chaperone network is highly efficient, misfolded proteins still aggregate due to various causes, such as genetic mutations and environmental and oxidative stresses. Disaggregation complexes are needed to resolve these aggregates. In yeast cells, the AAA+ ATPase Hsp104 is a powerful disaggregase that is recruited to protein aggregates with the assistance of Hsp70 and Hsp40 to facilitate disaggregation [12]. Recent studies have also implicated Hsp110 (Sse1) in facilitating protein disaggregation in both yeast and mammalian cells [13–15]. Depending on their misfolded state, disaggregated proteins are destined for either refolding or degradation [12]. Thus, disaggregase machineries are critical in protecting cells from the harmful effects of protein aggregation.

Accumulation of protein aggregates is a hallmark of several neurodegenerative diseases [16, 17]. Huntington’s disease (HD) is a dominant neurodegenerative disorder caused by a trinucleotide (CAG) repeat expansion in exon I of the *HTT* gene, which results in terminal misfolding and aggregation of Htt proteins. HD is often a late-onset disease which is likely attributed to decreased activity of protein quality control systems, including the ubiquitin proteasome system (UPS) [18–21]. In neuronal cells, less efficient UPS activity is linked to neurodegenerative diseases including HD [22]. Budding yeast has been used as a model organism to study the cellular response to the expression of misfolded human disease proteins. In yeast cells, overexpression of mutated Huntingtin with 103 polyQ expansion and the proline-rich domain (Htt103QP) results in aggregation, making it an ideal substrate to study the process of protein aggregation and clearance [23, 24]. Interestingly, overexpression of Htt103QP is not toxic to yeast cells because it is sequestered and deposited into cytoprotective inclusion bodies (IB) which can be cleared via autophagy [25–27]. However, the role of chaperone proteins in the clearance of mutated Huntingtin remains largely unexplored.

In this study, we identified a chaperone network that is required for Htt103QP IB formation and the subsequent autophagic degradation in yeast cells. The cytosolic Hsp70 Ssa chaperones, the Hsp70 NEFs Sse1 and Fes1, and Hsp40 co-chaperone Ydj1 are required for Htt103QP IB formation. Interestingly, in cells lacking Sse1 and Fes1, Htt103QP aggregation is accelerated. In addition, the aggregation of other proteins is also more pronounced as evidenced by the formation of Hsp104-GFP foci in the absence of Htt103QP expression. Surprisingly, *sse1Δ*, *fes1Δ* and *ydj1-151* mutants exhibit no significant delay in proteasome-dependent degradation of Htt103QP. However, we found that the recognition of Htt103QP aggregates by the autophagy machinery was much less efficient in *sse1*Δ, *fes1*Δ and *ydj1-151* mutants, as Htt103QP co-localization with the autophagosomal marker Atg8 was significantly reduced. As a result, the rate of autophagic degradation of Htt103QP was substantially decreased in these mutants. Therefore, our data support the conclusion that the absence of a particular group of HSPs results in abnormal aggregation of misfolded proteins, which compromises their clearance through the autophagy pathway.

## Results

### Sse1 and Fes1 are required for mutant Huntingtin inclusion body formation

**Fig 1:**
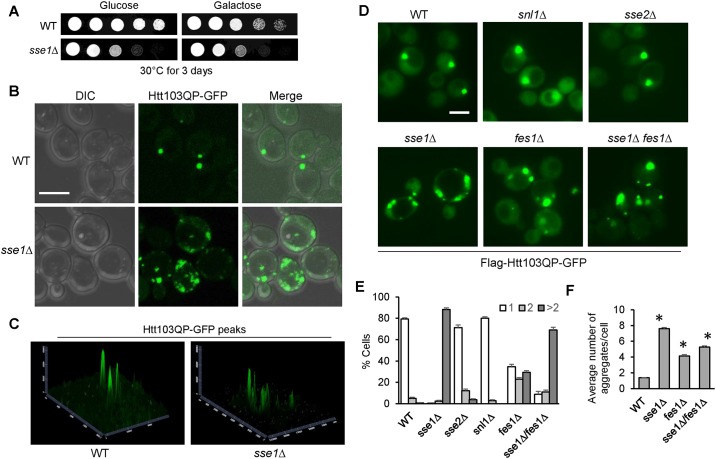
Cytosolic Hsp70 nucleotide exchange factors Sse1 and Fes1 are required for Htt103QP inclusion body (IB) formation. (A) Htt103QP overexpression and the growth of WT and *sse1*Δ cells. Cells containing *P*_*GAL*_*Flag-Htt103QP-GFP* were grown to saturation, 10-fold diluted, and spotted onto glucose (YPD) or galactose (YEPG) (yeast extract peptone and galactose) plates. The plates were incubated at 30°C for 2 days. (B) Confocal DIC and fluorescent images showing Htt103QP IB formation. WT and *sse1*Δ cells with *P*_*GAL*_*Flag-Htt103QP-GFP* were incubated at 30°C in YEPG medium for 16 hrs to induce Htt103QP-GFP expression. The cells were fixed by paraformaldehyde for 5 min and then resuspended in PBS buffer for confocal microscopy. Scale bar = 5μm. **(C)** 2.5-D analysis showing the intensity of fluorescent GFP peaks of the images from (B). Software used was Zen Blue from Zeiss. (D) Htt103QP IB formation in yeast mutants lacking Hsp70 nucleotide exchange factors (NEFs). Cells with the indicated genotypes were grown and treated as described above and then examined using an EVOS fluorescence microscope. Representative fluorescent images are shown for WT, *snl1*Δ, *sse1*Δ, *sse2*Δ, *fes1*Δ, and *sse1*Δ *fes1*Δ cells expressing Htt103QP-GFP. Scale bar = 5μm. (E) Cells from (D) were quantified for the number of aggregates in each cell: 1, 2 or >2 (n = 100 cells). The results are the average of three independent experiments. (F) The average number of aggregates in WT, *sse1*Δ, *fes1*Δ, and *sse1*Δ *fes1*Δ cells from (D) were quantified (n > 40 cells). The results are the average of three independent experiments. * indicates statistical comparison between WT and mutants. p < .0001 in all instances.

Inclusion body (IB) formation is an active cytoprotective process that sequesters misfolded protein species [25–28]. In budding yeast *Saccharomyces cerevisiae*, overexpression of mutant Huntingtin with 103 polyQ repeat and the proline-rich domain (Htt103QP) from a galactose promoter (*P*_*GAL*_*Flag-Htt103QP-GFP*) leads to IB formation [23, 24]. However, many of the components necessary for IB formation have yet to be discovered. From a genome-wide genetic screen, we identified *dsk2*Δ mutant that showed compromised IB formation and was sensitive to Htt103QP expression [26]. From this screen, we also found that *sse1*Δ mutant exhibited slow growth on galactose plates, but this slow growth phenotype was independent of Htt103QP overexpression, as the mutant cells grew slowly on both galactose and glucose plates (Fig 1A). A previous study found that Sse1 associates with Htt103QP aggregates formed in yeast cells [25], therefore, we examined Htt103QP IB formation in *sse1*Δ mutant. For this experiment, cells were incubated in galactose medium for 16 hrs to induce Htt103QP-GFP overexpression. The majority of wild-type (WT) cells exhibited one single large inclusion body marked by GFP, but *sse1*Δ cells had multiple Htt103QP aggregates, which were often smaller and morphologically different from the inclusions in WT cells (Fig 1B). Moreover, the GFP intensity of the aggregates in *sse1*Δ cells was considerably less than those in WT cells (Fig 1C). We found that majority of WT cells (79%) had only one IB, whereas 88% of *sse1*Δ mutant cells exhibited three or more aggregates, with an average around eight aggregates in a *sse1*Δ cell (Fig 1E and 1F). Therefore, *sse1*Δ mutant cells show an increased number of aggregates and exhibit a clear IB formation defect.

Sse1 is one of the four cytosolic Hsp70 nucleotide exchange factors (NEF) in yeast cells, with the other three being Sse2, Fes1 and Snl1 [9]. Therefore, we examined if the others were also required for Htt103QP IB formation. *sse2*Δ and *snl1*Δ mutant cells exhibited normal IB formation after Htt103QP induction, but *fes1*Δ mutant showed a clear IB formation defect as indicated by the increased number of GFP foci, albeit less pronounced than *sse1*Δ cells (Fig 1D). Approximately 35% of *fes1*Δ mutant cells were still able to form a single large IB like WT cells, but other cells contained additional smaller aggregates (Fig 1D and 1E). This is consistent with the average number of aggregates in *fes1*Δ cells being more than WT but less than *sse1*Δ cells (Fig 1F). Since both *sse1*Δ and *fes1*Δ mutants exhibited IB formation defect, we speculated that a double mutant might exacerbate the phenotype. Interestingly, the *sse1*Δ *fes1*Δ double mutants did not show exacerbated phenotype, but instead exhibited a phenotype more similar to *fes1*Δ cells (Fig 1D, 1E and 1F). This might be attributed to the dramatic increase in heat shock response in *sse1*Δ *fes1*Δ double mutants [9], as enhanced expression of other HSPs may suppress the severity of the *sse1*Δ *fes1*Δ phenotype. Taken together, these results indicate that cytosolic Hsp70 NEF Sse1, and Fes1 to a lesser extent, participates in IB formation in yeast cells overexpressing Htt103QP.

### Deletion of either *SSE1* or *FES1* results in accelerated Htt103QP aggregation

**Fig 2:**
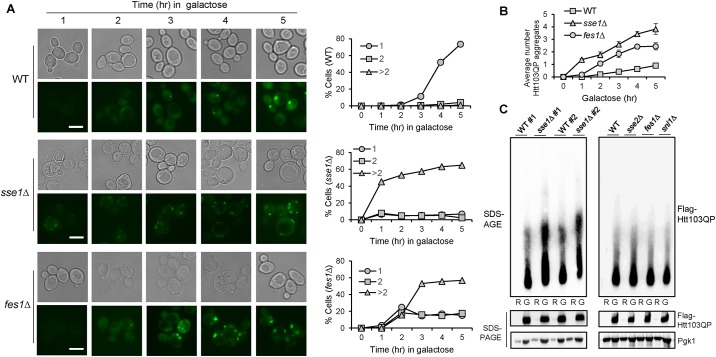
Cells lacking Sse1 and Fes1 exhibit accelerated Htt103QP aggregate formation. (A) WT, *sse1*Δ, and *fes1*Δ cells with *P*_*GAL*_*Flag-Htt103QP-GFP* plasmid were grown in non-inducible raffinose medium (YEP + Raffinose) to mid log phase, and then galactose was added to induce the overexpression of Flag-Htt103QP-GFP. Cells were collected every hour for microscopy using the EVOS microscope. Representative DIC and fluorescence images are shown for Htt103QP aggregate formation. Scale bar = 5μm. The number of aggregates in each cell were quantified. The percentage of cells with 1, 2 or >2 aggregates (n = 100 cells) is shown. (B) Quantitation of the average number of aggregates per cell in WT, *sse1*Δ and *fes1*Δ cells. Averages are from three independent experiments (n > 50 cells). (C) Detection of Htt103QP protein aggregates using agarose gel electrophoresis. WT and mutant cells were grown in non-inducible raffinose medium (R). After galactose addition, the cells were incubated for 3 hr (G). Protein samples were prepared as described in Materials and Methods and subjected to SDS agarose gel electrophoresis (SDS-AGE) and SDS-PAGE. Two separate WT and *sse1*Δ strains are shown for reproducibility (left). The results for WT, *sse2*Δ, *fes1*Δ, and *snl1*Δ mutants are shown in the right panel. Pgk1: loading control.

Sse1 has previously been implicated in preventing protein aggregation by assisting in protein folding and facilitating protein disaggregation [15, 29, 30]. In this context, we tested whether *sse1*Δ and *fes1*Δ mutants changed the rate of aggregation after Htt103QP overexpression. Cells with *P*_*GAL*_*Flag-Htt103QP-GFP* were grown to mid-log phase in non-inducible raffinose containing medium, and then galactose was added into the medium to induce Htt103QP overexpression. Cells were collected over time to visualize the appearance of GFP foci, which indicate Htt103QP aggregate formation. We found that both *sse1*Δ and *fes1*Δ mutants exhibited accelerated aggregation of Htt103QP compared to WT cells (Fig 2A). In cells lacking Sse1 and Fes1, visible aggregates appeared at 1 and 2 hr after Htt103QP induction, respectively, while visible aggregates began to appear in WT cells after 3 hr induction. Furthermore, the average number of aggregates was substantially higher in both mutants with *sse1*Δ mutants averaging nearly four per cell after 5 hr galactose induction (Fig 2B). To validate these microscopic observations biochemically, we performed SDS agarose gel electrophoresis (SDS-AGE) [31]. Htt103QP species with higher molecular weight were observed in *sse1*Δ mutants compared to WT after 3 hr galactose induction, suggesting more pronounced Htt103QP aggregation in *sse1*Δ cells (Fig 2C). As expected, increased aggregated Htt103QP was not observed in *sse2*Δ and *snl1*Δ mutant cells. Surprisingly, we did not observe an appreciable increase in Htt103QP aggregates in *fes1*Δ cells using SDS-AGE (Fig 2C), indicating that the biochemical nature of the aggregates observed in *sse1*Δ and *fes1*Δ is likely different. These results suggest that Sse1 and Fes1 aid in preventing the aggregation of Htt103QP in yeast cells, with Sse1 seemingly playing a more predominant role.

### Hsp70 and Hsp40 chaperones facilitate Htt103QP IB formation

**Fig 3:**
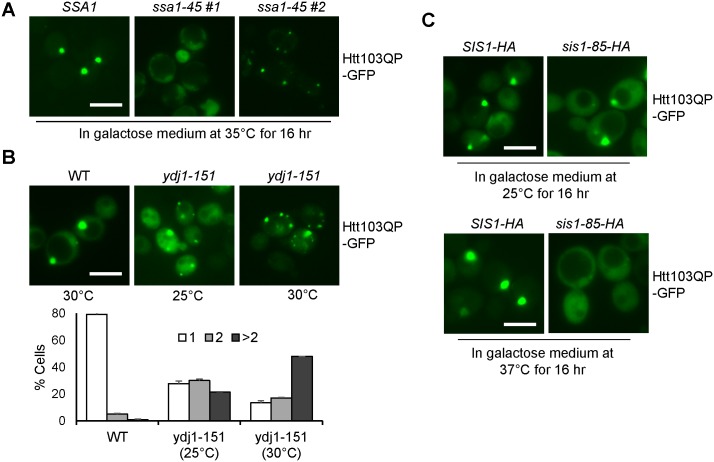
Hsp70 and Hsp40 chaperones participate in Htt103QP IB formation. (A) Htt103QP IB formation in yeast Hsp70 Ssa family mutants. Fluorescent images of *SSA1-WT* and *SSA1-45* cells expressing Flag-Htt103QP-GFP at 35°C for 16 hr. Two separate fluorescent images for *ssa1-45* mutants indicate two phenotypes. Scale bar = 5μm. (B) Htt103QP IB formation in Hsp40 co-chaperone mutant *ydj1-151*. Fluorescent images of WT and *ydj1-151* cells expressing Flag-Htt103QP-GFP for 16 hrs at permissive (25°C) and semi-permissive (30°C) temperatures. Cells were quantified for the number of aggregates in each cell: 1, 2 or >2 (n = 100 cells). The results are the average of three independent experiments. Scale bar = 5μm. (C) Htt103QP IB formation in Hsp40 co-chaperone mutant *sis1-85*. Fluorescent images of *SIS1-HA* and *SIS1-85-HA* strains expressing Htt103QP-GFP for 16 hrs at permissive (25°C) and restrictive (37°C) temperatures. Scale bar = 5μm. All microscopy in this figure was performed on the EVOS microscope.

Sse1 and Fes1 are NEFs that replace ADP with ATP on the cytosolic Ssa subfamily of Hsp70 [32, 33]. This subfamily includes Ssa1, Ssa2, Ssa3 and Ssa4, and they interact with both Sse1 and Fes1 [34]. Therefore, we determined whether these chaperones played a role in Htt103QP IB formation. We first examined the IB formation in each single deletion mutant, but no obvious defect was observed (S1A Fig). We speculated the efficient Htt103QP IB formation in each single mutant is likely due to the redundant function of Hsp70 protein family. To test this possibility, we used a strain lacking *SSA2*, *SSA3* and *SSA4* genes but expressing temperature-sensitive *ssa1-45* [35]. We found that this mutant showed a significant Htt103QP IB formation defect when incubated at 35°C. Many of the mutant cells exhibited diffuse Htt103QP-GFP, while others showed numerous small aggregates (Fig 3A). Due to the diffuse nature of Htt103QP in many mutant cells, quantitation was not performed. These results indicate that cytosolic Hsp70 proteins are critical for Htt103QP IB formation.

We next examined the role of Hsp40 chaperones in Htt103QP IB formation. Hsp40 co-chaperone proteins stimulate the ATPase activity of Hsp70 to facilitate complex formation between Hsp70 and client proteins [11]. Previous work indicates that Hsp40 co-chaperone proteins Sis1 and Ydj1 physically interact with Sse1 [34]. Therefore, we examined IB formation in the absence of Hsp40 co-chaperones Ydj1 and Sis1. Because *YDJ1* deletion mutant is very sick and *SIS1* is an essential gene, we used temperature sensitive mutants for this experiment [36, 37]. The non-permissive temperature of *ydj1-151* strain is 37°C, but multiple Htt103QP aggregates were observed in the mutant cells even when incubated at 25°C, and the phenotype was further exacerbated at 30°C (Fig 3B). The temperature sensitive *sis1-85* mutant cells failed to aggregate any Htt103QP at non-permissive 37°C (Fig 3C), which is likely attributable to its function in [RNQ+] maintenance, as [RNQ+] is essential for the aggregation of mutant Huntingtin proteins in yeast cells [37, 38]. Therefore, Ydj1 co-chaperone is required for Htt103QP IB formation.

In addition to the Ssa family, other chaperones are also known to interact with either Sse1 or Fes1, such as Ssb1, Sti1, Hsp42, Hsp82 and Hsc82 according to the *Saccharomyces* genome database. We examined the Htt103QP IB formation in yeast mutants lacking these proteins, none of which exhibited an obvious defect in IB formation (S1C Fig). Taken together, these results suggest that a chaperone network is required to facilitate Htt103QP IB formation, and this network includes the Ssa class of Hsp70s, NEFs Sse1 and Fes1, and the Hsp40 co-chaperone Ydj1.

### Analysis of protein aggregation in HSP mutants using disaggregase Hsp104 as a marker

**Fig 4:**
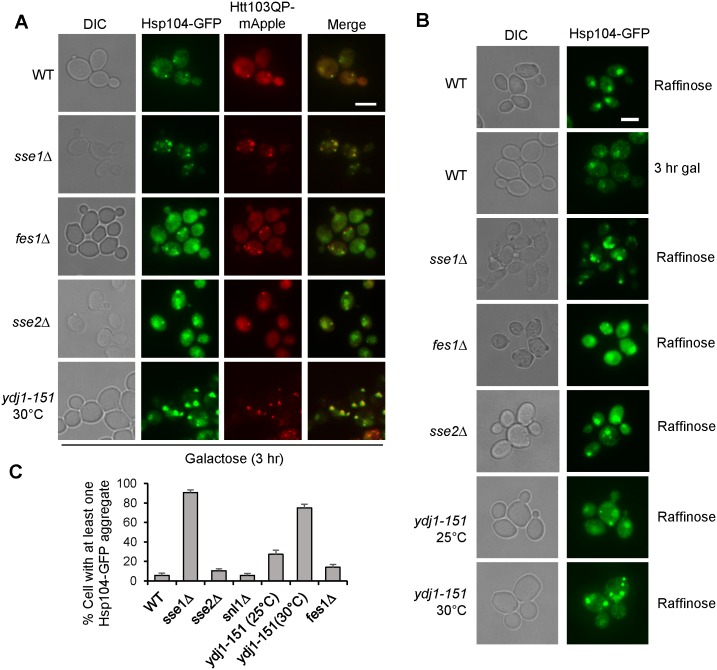
Hsp104-GFP positive aggregates are increased in *sse1*Δ and *ydj1-151* mutants. (A) Hsp104 recruitment to Htt103QP aggregates is independent of chaperones. Cell with indicated genotypes were grown in non-inducible raffinose medium to mid-log phase then galactose was added. Images were taken after incubation for 3 hr. Representative fluorescence images show cellular localization of Hsp104-GFP and Htt103QP-mApple. DIC and merged images are also shown. Scale bar = 5μm. (B) Constitutive appearance of Hsp104-GFP positive aggregates in *sse1*Δ and *ydj1-151* mutants. Cells with indicated genotypes were grown in raffinose or galactose containing medium to mid-log phase. Representative fluorescence images are shown. WT cells show predominantly nuclear localized Hsp104-GFP in the absence of Htt103QP overexpression. Scale bar = 5μm. (C) Cells from (B) were quantified based on the presence of Hsp104-GFP positive aggregate(s). Results are the average of three independent experiments (n = 100 cells). All images in this figure was obtained using the EVOS microscope.

The function of the yeast AAA+ ATPase Hsp104 in protein disaggregation is well established [12]. Previous studies have demonstrated that Hsp104 functions with Hsp40 and Hsp70 to facilitate protein disaggregation [39, 40], whereas more recent work indicates that Hsp110 (Sse1 and Sse2) aids Hsp104 in disaggregation of protein aggregates [13, 41]. Therefore, we determined whether Sse1 and Fes1 recruit Hsp104 to Htt103QP aggregates for disaggregation. For this experiment, WT, *sse1*Δ, and *fes1*Δ cells with *HSP104-GFP P*_*GAL*_*Flag-Htt103QP-mApple* were grown in galactose medium for 6 hr to induce Htt103QP overexpression. Surprisingly, the co-localization of Hsp104-GFP and Htt103QP-mApple was similar in WT, *sse1*Δ and *fes1*Δ cells (Fig 4A), even though the mutants contain more aggregates. This observation indicates that NEFs Sse1 and Fes1 are not necessary for Hsp104 to recognize Htt103QP aggregates. We also examined the colocalization of Hsp104-GFP with Htt103QP-mApple in *sse2*Δ and *ydj1-151* mutants, no colocalization defect was observed, which is similar to *sse1*Δ and *fes1*Δ mutants (Fig 4A). Although Hsp104 seems to recognize Htt103QP aggregates efficiently in these mutants, further biochemical studies are needed to determine the disaggregase functionality of Htt103QP-associated Hsp104 in these mutants.

When examining the Hsp104 –Htt103QP colocalization, WT cells showed enriched nuclear localization of Hsp104-GFP with very few cytoplasmic GFP foci prior to Htt103QP induction (S2 Fig). The observation regarding the enriched Hsp104-GFP nuclear localization under non-stressed conditions differs from the findings from the Glover lab [42], but is consistent with findings from the Wendland lab [43]. These differences are most likely attributable to N-terminal (the Glover lab) vs C-terminal GFP tagging of Hsp104 (the Wendland lab and this study). Strikingly, cytoplasmic Hsp104-GFP foci were observed in *sse1*Δ and *ydj1-151* mutants, and to a lesser extent *fes1*Δ, even before Htt103QP induction, indicating aggregate formation (Fig 4B). For example, 91% *sse1*Δ mutant cells contained at least one cytoplasmic Hsp104-GFP positive foci. These foci were also observed in *ydj1-151* mutant even at permissive temperature 25°C and drastically increased at 30°C (Fig 4B and 4C). This phenomenon was not observed in *sse2*Δ cells, which is consistent with the normal Htt103QP IB formation in these cells. Taken together, these results suggest that Sse1, Fes1 and Ydj1 are chaperones critical to prevent protein aggregation and/or promote aggregate disassembly.

### Chaperones play a minor role in proteasomal degradation of Htt103QP

Mutated Huntingtin with polyQ expansion can be degraded via both UPS and autophagy pathways [44]. We speculated that the accelerated aggregation of Htt103QP in some HSP mutants could be a result of compromised protein degradation. Therefore, we examined proteasome-dependent degradation of Htt103QP in *sse1*Δ and *fes1*Δ mutant cells. We have previously shown that Htt103QP is efficiently degraded via the proteasome using a shut-off assay [26]. Here, we confirmed the efficient degradation of Htt103QP after 1 hr induction in galactose medium followed by expression shut-off by adding glucose. Inhibition of proteasome activity with MG132 blocked Htt103QP degradation (S3A Fig). In contrast, deletion of autophagy genes, *atg7*Δ and *atg8*Δ, had little effect on the degradation Htt103QP using the shut-off assay (S3B Fig). Therefore, the rapid disappearance of Htt103QP protein in WT cells after short-time induction depends on the UPS, but not the autophagy pathway. We next examined proteasome-dependent degradation of Htt103QP in yeast mutants lacking Hsp70 NEFs. Interestingly, we observed no significant delay in proteasomal degradation of Htt103QP in NEF mutants, *sse1*Δ, *fes1*Δ, *sse2*Δ, and *snl1*Δ (S3C and S3D Fig). In addition, we found no obvious delay in proteasomal degradation of Htt103QP in *ydj1-151* or *sis1-85* mutants at restrictive temperature 37°C (S3E Fig). Sis1 has been shown to be required for the proteasomal degradation of endogenous misfolded proteins when fused with a degron sequence from Ndc10 [45]. Since Htt103QP is expressed as a misfolded protein without this degron sequence, we speculate that Sis1 plays distinct roles for the degradation of different substrates. Similarly, no obvious Htt103QP degradation defect was observed in mutant cells lacking other chaperone proteins known to interact with Sse1 (S1B and S1D Fig). The efficient Htt103QP degradation in *sse1*Δ, *fes1*Δ, and *ydj1-151* mutants is surprising, given the accelerated Htt103QP aggregation observed in these mutants. To test the possibility that this proteasome-mediated degradation of Htt103QP could only be for the soluble fraction, we performed a sedimentation as previously described [46]. We found that after 2 hr induction nearly all of the Htt103QP proteins still remained soluble in both WT and *sse1*Δ mutants (S4A Fig). We speculate that the Htt103QP aggregates observed in *sse1*Δ mutant cells after short-time induction could be soluble, which enables efficient proteasomal degradation of Htt103QP.

Protein ubiquitination is a targeting signal for proteasome-dependent degradation [47]. Previous studies indicate that both Sse1 and Fes1 are involved in Hsp70-dependent ubiquitination and degradation of misfolded proteins [48–52]. Because the proteasomal degradation was normal in these mutants, we reasoned that Htt103QP ubiquitination should not be compromised in these mutants. We examined Htt103QP ubiquitination in *sse1*Δ mutant as it exhibited the most severe phenotype. As expected, *sse1*Δ mutant exhibited similar Htt103QP ubiquitination compared to WT cells (S4B Fig). These results indicate that while some chaperone proteins prevent the rapid aggregation Htt103QP, they are not required for its ubiquitination and efficient proteasomal degradation.

### Autophagic degradation of Htt103QP is impaired in *sse1*Δ, *fes1*Δ and *ydj1-151* mutant cells

**Fig 5:**
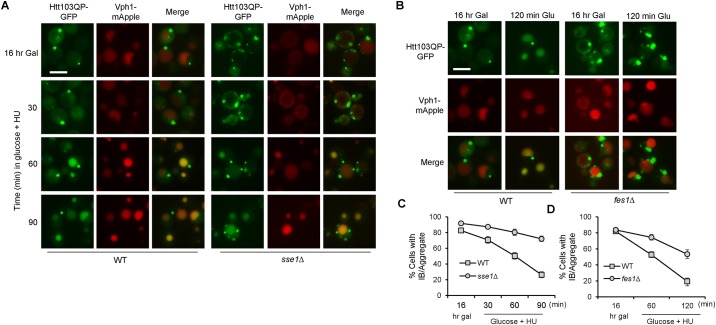
The autophagic degradation of Htt103QP is compromised in *sse1*Δ and *fes1*Δ mutants. (A) The autophagic disposal of Htt103QP in WT and *sse1*Δ mutant cells. *pep4*Δ *VPH1-mApple* (WT) and *sse1*Δ *pep4*Δ *VPH1-mApple* (*sse1*Δ) cells containing Flag-Htt103QP-GFP were grown in galactose medium (YEPG) at 30°C for 16 hr. Glucose was then added to shut off Htt103QP expression, and 100 mM hydroxyurea (HU) was added to block cell cycle progression. Representative fluorescent images are shown for the localization of Htt103QP-GFP and Vph1-mApple before and after addition of glucose and HU. Vph1-mApple was used to mark vacuole structure. Pep4 is a vacuolar protease, and *pep4*Δ mutant was used to slow degradation inside the vacuole to enhance visualization. (B) Htt103QP autophagic disposal in WT and *fes1*Δ mutant cells. A similar protocol was used to examine the vacuolar localization of Htt103QP-GFP. (C and D) Quantitation of cells with Htt103QP IB or aggregates localized outside the vacuole over time. The cells from (A) and (B) are used for the quantitation. The quantified results (three independent repeats) were obtained by counting the number of cells containing at least one IB or aggregate (n = 100). All microscopy in this figure was performed on the EVOS microscope.

Previous studies have analyzed the roles of chaperone proteins in IB formation and alleviation of toxic effects of aggregation-prone proteins [22, 53–56]. The chaperone-mediated autophagy (CMA) pathway has been shown to target soluble cytosolic proteins containing KFERQ-like-motifs for autophagic degradation in mammalian cells [57]. However, very few studies have directly analyzed the role of chaperone proteins in the autophagic degradation of mutant Huntingtin protein. We recently showed that Htt103QP is efficiently degraded via autophagy in yeast cells [26]. Thus, we used a similar protocol to analyze this process in *sse1*Δ and *fes1*Δ mutant cells. Htt103QP-GFP expression was induced in galactose medium for 16 hr, then glucose and DNA synthesis inhibitor hydroxyurea (HU) were added to the medium to shut off Htt103QP-GFP expression and prevent cell division, respectively. After glucose and HU were added for 30 min, vacuolar GFP localization was observed in some WT cells, indicating the transport of Htt103QP-GFP into the vacuole via the autophagy pathway. After 90 min incubation, only 26% of WT cells still contained Htt103QP aggregate(s), but 72% of *sse1*Δ cells contained aggregates (Fig 5A and 5C). Therefore, we conclude that *sse1*Δ mutant cells exhibit a significant autophagic degradation defect for Htt103QP. We also found that *fes1*Δ mutants exhibited impaired autophagic degradation of Htt103QP. After glucose and HU were added for 120 min, 53% of *fes1*Δ cells showed Htt103QP-GFP aggregates, compared to 19% in WT cells (Fig 5B and 5D). These results indicate the critical role of chaperone proteins in the efficient autophagic clearance of misfolded proteins.

**Fig 6:**
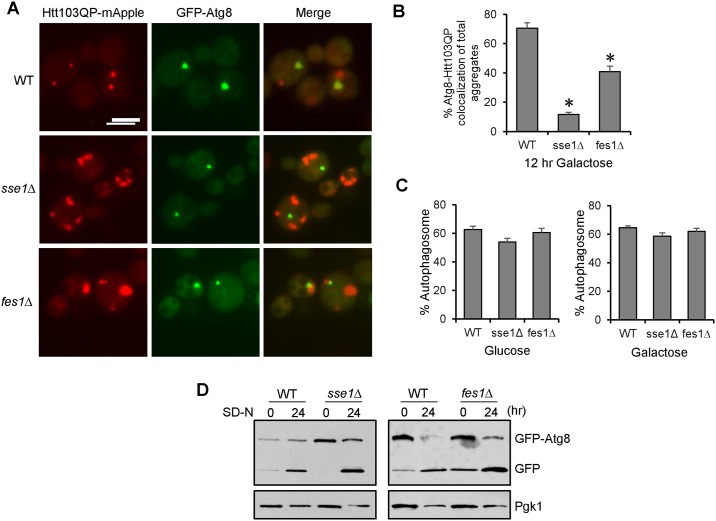
Sse1 and Fes1 are required for efficient recognition of Htt103QP by autophagy machinery. (A) The co-localization of Htt103QP and Atg8 in WT, *sse1*Δ, and *fes1*Δ cells. Cells containing *GFP-ATG8* and *P*_*GAL*_*Flag-Htt103QP-mApple* were grown in galactose medium YEPG for 16 hrs and fixed for microscopy. Representative fluorescence images show the localization of Htt103QP-mApple and GFP-Atg8. Scale bar = 5μm. (B) Quantitation of total number of Htt103QP aggregates colocalized with GFP-Atg8 in WT, *sse1*Δ, and *fes1*Δ cells (n = 100 IB/aggregates). Quantities indicate the average of three independent experiments. * indicates statistical comparison. p < .001. (C) Quantitation of the number of GFP-Atg8 autophagosomes present in WT, *sse1*Δ and *fes1*Δ cells incubated in glucose or galactose medium (n = 100 cells). Quantities indicate the average of three independent experiments. (D) Sse1 and Fes1 are not required for starvation-induced autophagy. WT, *sse1*Δ, and *fes1*Δ cells with *GFP-ATG8* were grown in SC -TRP medium, and then shifted to nitrogen-deficient medium (SD-N) for 24 hr. The cells were collected to detect the presence of GFP-Atg8 and the cleavage product, monomeric GFP, using anti-GFP antibody. Pgk1: loading control. The EVOS microscope was used to obtain the images in this figure.

Aggregated proteins can be degraded through selective autophagy, which involves the recognition and sequestration of the cargo within the autophagosome, a key structure in autophagy. An autophagosome is a double membrane vesicle that delivers cytoplasmic components to lysosomes/vacuoles. Atg8 is a protein anchored to the autophagosomal membrane and is required for autophagosome formation [58]. We speculated that the selective autophagy machinery may not be able to efficiently recognize the Htt103QP aggregates in *sse1*Δ and *fes1*Δ mutants, which could contribute to the defect in clearance. To test this possibility, we examined the co-localization of Htt103QP with Atg8. For this purpose, we introduced a plasmid expressing *GFP-ATG8* into yeast cells with *P*_*GAL*_*Flag-Htt103QP-mApple*, and the cells were incubated in galactose medium for 16 hr to induce Htt103QP overexpression. Indeed, we found a substantial decrease in the co-localization of Htt103QP-mApple aggregates with GFP-Atg8 in *sse1*Δ and *fes1*Δ mutants (Fig 6A). The percentage of total Htt103QP aggregates that co-localize with Atg8 was 11% in *sse1*Δ cells compared to 70% in WT cells (Fig 6B). Importantly, this decrease was not due to defects in autophagosome formation in the mutants, as the frequency of visible autophagosomes (GFP-Atg8 foci) was similar in WT and *sse1*Δ cells incubated in galactose and glucose media (Fig 6C). In addition, starvation-induced non-selective autophagy was normal, and possibly even more efficient, in *sse1*Δ and *fes1*Δ mutants as indicated by the production of monomeric GFP generated from GFP-Atg8 cleavage after starvation [59] (Fig 6D). Taken together, these results indicate that Sse1 and Fes1 promote autophagy-mediated degradation of Htt103QP by allowing the effective recognition of the aggregates by the selective autophagy machinery.

**Fig 7:**
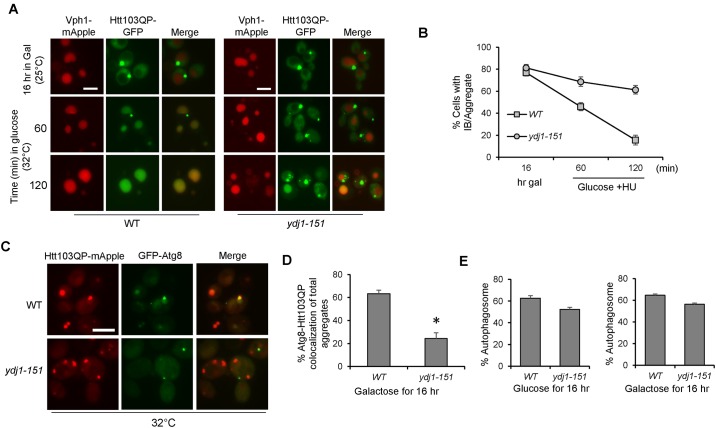
Hsp40 co-chaperone Ydj1 is required for efficient autophagic degradation of Htt103QP. (A) Htt103QP autophagy in WT and *ydj1-151* mutant cells. *pep4*Δ *VPH1-mApple* (WT) and *ydj1-151 pep4*Δ *VPH1-mApple* (*ydj1-151*) cells containing *P*_*GAL*_*Flag-Htt103QP-GFP* were grown in galactose medium (YEPG) at 25°C for 16 hrs. Cells were then shifted to semi-permissive temperatures (32°C) for 10 min prior to glucose and hydroxyurea (HU) addition. Representative fluorescent images are shown for the localization of Htt103QP-GFP and Vph1-mApple (vacuolar marker) before and after addition of glucose and HU. Scale bar = 5μm. (B) Quantitation of cells with Htt103QP IB or aggregates localized outside the vacuole over time. Results from three independent experiments were used for the quantitation. The quantified results were obtained by counting the number of cells containing at least one IB or aggregate (n = 100). (C) The co-localization of Htt103QP and Atg8 in WT and *ydj1-151* cells. Cells containing *GFP-ATG8* and *P*_*GAL*_*Flag-Htt103QP-mApple* were grown in galactose medium YEPG for 16 hrs at 32°C and fixed for microscopy. Representative fluorescence images show the localization of Htt103QP-mApple and GFP-Atg8. (D) Quantitation of total number of Htt103QP aggregates colocalized with GFP-Atg8 in WT and *ydj1-151* cells (n = 100 IB/aggregates). Quantities indicate the average of three independent experiments. * indicates statistical comparison. p < .005. (E) Quantitation of the number of GFP-Atg8 autophagosomes present in WT and *ydj1-151* cells incubated in glucose or galactose medium (n = 100 cells). Quantities indicate the average of three independent experiments. All images in this figure were acquired using the EVOS microscope.

Since *ydj1-151* mutants exhibit similar IB formation defects as *sse1*Δ and *fes1*Δ mutants, we next examined if *ydj1-151* cells exhibited an autophagy defect as well. For this experiment, Htt103QP expression was induced in WT and *ydj1-151* cells in galactose medium at 25°C for 16 hrs, then the cultures were shifted to semi-permissive temperature 32°C for 15 minutes before addition of glucose and HU to shut off Htt103QP expression and block cell cycle progression, respectively. After glucose addition for 120 min, only 16% of WT cells contained Htt103QP aggregates and most of the cells showed obvious GFP signal in the vacuole, indicating successful autophagy. In *ydj1-151* mutants, however, 61% of cells contained at least one Htt103QP aggregate, and the vacuolar GFP signal was much less frequent (Fig 7A and 7B). Consistent with the lack of vacuole localization, the frequency of Htt103QP aggregates colocalizing with GFP-Atg8 was dramatically reduced in *ydj1-151* mutant at semi-permissive temperature 32°C (Fig 7C and 7D). The frequency of visible GFP-Atg8 autophagosomes in WT and *ydj1-151* mutant cells incubated in galactose or glucose media was similar, indicating that the autophagic degradation defect in *ydj1-151* mutant was not due to defective autophagosome formation (Fig 7E). Taken together, these results indicate that chaperone proteins Sse1, Fes1, and Ydj1 are required for the recognition of Htt103QP aggregates by Atg8 and the subsequent autophagosome formation. Therefore, cells lacking these chaperones show a significant defect in autophagic clearance of Htt103QP.

## Discussion

The accumulation of misfolded proteins in neurons is a hallmark of neurodegenerative diseases [60, 61]. The stressful environment caused by protein aggregates may interfere with cellular functions. The chaperone network prevents aggregation and facilitates refolding [5]. Thus, chaperone expression alterations are associated with aging and neurodegeneration [53]. The role of chaperones in protein folding, disaggregation and proteasome-dependent degradation has been well documented [5, 12], but the role of chaperone proteins in autophagy-dependent clearance of misfolded proteins is not fully understood. Using yeast as a model system, we found that cytosolic Hsp70 proteins and their co-factors, Hsp40 co-chaperone and NEFs, are required for the efficient clearance of mutated Htt103QP through autophagy. Moreover, this defect is likely due to the failure of IB formation, as evidenced by the accelerated protein aggregation and the increased number of aggregates in the mutant cells.

The rapid accumulation of Htt103QP aggregates in *sse1*Δ, *fes1*Δ, and *ydj1-151* mutant cells is unlikely attributable to impaired Htt103QP degradation by the proteasome. All tested chaperone mutants exhibited proteasome-dependent degradation, although we found slight delay in some strains. Since polyubiquitination is a targeting signal for proteasome-dependent degradation and mutant Huntingtin protein is known to be ubiquitinated [27, 62, 63], we examined the ubiquitination state of Htt103QP in *sse1*Δ cells. The ubiquitination of Htt103QP was unaffected by *sse1*Δ deletion, arguing against the role of Sse1 in Htt103QP ubiquitination. We further tested the possibility that Sse1 and Fes1 recruit disaggregase Hsp104 to Htt103QP aggregates [64], but we found clear co-localization of Hsp104 with Htt103QP in *sse1*Δ and *fes1*Δ mutants. One explanation is that the Hsp104 is not fully functional in these mutant cells even though its recruitment to Htt103QP aggregates is normal. Alternatively, Sse1, Fes1, and Ydj1 chaperones could have a disaggregase function independent of Hsp104.

We have previously shown that Htt103QP can be efficiently cleared via autophagy [26]. We found that Sse1, Fes1, and Ydj1 are all required for efficient autophagy of Htt103QP. The rate of clearance of the multiple aggregates observed in these mutants is significantly delayed compared to WT cells. However, Htt103QP is still observed in the vacuole in some mutant cells, indicating autophagic degradation of Htt103Qp is not abolished completely. We found a stark decrease in the colocalization of autophagosmal marker Atg8 with Htt103QP aggregates in all three mutants. It is possible that the biochemical nature of the aggregates in these mutants differs from the IB in WT cells, and thus cannot be efficiently recognized and encapsulated into autophagosomes [65]. Another possibility is that the accelerated Htt103QP aggregation may block the binding sites for an autophagy receptor. Recent studies suggest that the ubiquitin-Atg8 adaptor Cue5 acts as the receptor to facilitate autophagy-dependent clearance of mutated Huntingtin [63]. Further studies are needed to examine the binding of Htt103QP to Cue5 in cells lacking HSPs.

It is of interest that the IB formation defects found in yeast cells lacking Sse1 and Fes1 do not cause cytotoxicity like in other mutants, such as *dsk2*Δ and *cdc48-1* and *bmh1*Δ [25, 26, 66]. One explanation is that the aggregates formed in *sse1*Δ and *fes1*Δ mutants are less toxic [67, 68]. However, the aberrant aggregates in *sse1*Δ mutants appear morphologically similar to the aggregates in cells overexpressing Htt103Q that lacks the proline-rich domain, and yeast cells are very sensitive to Htt103Q expression [38]. Our explanation is that *sse1*Δ mutant cells accumulate multiple aggregates even in the absence of Htt103QP expression as indicated in Fig 4, which may contribute to the slow growth of *sse1*Δ cells. Htt103QP expression does not further exacerbate the growth defect in *sse1*Δ cells.

Chaperone proteins are highly conserved from yeast to human. Our findings suggest that a chaperone network regulates aggregation of mutated Huntingtin. On one hand, these chaperone proteins prevent aggregation of misfolded proteins after their synthesis. On the other hand, this network is required for IB formation and the subsequent autophagic clearance. Therefore, functional HSPs are critical for the healthy cellular environment, and dysfunctional HSPs may contribute to disease development. In line with this, transgenic expression of Hsp110 is linked to extended survival in mice with ALS [69]. In contrast, loss of Hsp110 is linked to early accumulation of Alzheimer’s disease proteins Aβ and hyperphosphorylated Tau in mice [70]. Additionally, Hsp70 is known to bind and facilitate refolding of mutant Huntingtin, α-synuclein, Aβ, hyperphosphorylated Tau and mutant SOD1 [22]. Further mechanistic insights into this pathway may uncover potential therapeutic strategies.

## Materials and methods

### Strains, plasmids and growth conditions

Yeast strain used in this study are W303 unless otherwise noted. Genotypes are listed in S1 Table. Gene deletions and GFP tagging of *HSP104* were performed using a PCR-based method [71]. The plasmid of Flag- and GFP-tagged Htt103QP fragment with galactose-inducible promoter (*P*_*GAL*_*Flag-Htt103QP-GFP*) was originally from the Lindquist lab [23]. Using this plasmid, we subcloned the *P*_*GAL*_*Flag-Htt103QP-GFP* into pRS406 vector, and the resulting plasmid was then inserted into the yeast genome after EcoRV digestion. To construct the *P*_*GAL*_*Flag-Htt103QP-mApple* plasmid, the GFP was replaced with mApple using PCR-based subcloning. The pRS414-GFP-Atg8 plasmid, originally from the Klionsky lab [72], was obtained from Addgene. The p1217 plasmid is a derivative of pRS414 containing a galactose inducible promoter followed by 3xHA. We used PCR-based protocol to subclone the WT ubiquitin sequence from pRK5-HA-Ub [73] into p1217 to obtain *P*_*GAL*_*HA-Ub* plasmid. Yeast extract/peptone medium supplied with raffinose, galactose or glucose was used for the growth of yeast strains, except for those carrying centromeric plasmids.

### Fluorescence imaging

Confocal fluorescence imaging was performed using a Zeiss LSM 880 microscope (Oberkocken, Germany). All other fluorescence imaging analyses were performed using an EVOS microscope (Thermo Fisher Scientific, Waltham, MA). For all imaging analysis, samples were fixed in paraformaldehyde for 5 minutes then resuspended in 1× PBS buffer.

### Western blotting

Protein samples were prepared using an alkaline method and resolved by 10% SDS–PAGE. Anti-Flag antibody was purchased from Sigma-Aldrich (St. Louis, MO); anti-GFP antibody was from Santa Cruz Biotechnology (Santa Cruz, CA); anti-Pgk1 antibody was from Molecular Probes (Eugene, OR); anti-HA antibody was from Covance Research Products (San Diego, CA). The horseradish peroxidase–conjugated goat anti-mouse IgG secondary antibody was from Santa Cruz Biotechnology (Santa Cruz, CA).

### Starvation induced GFP-Atg8 cleavage assay

Cells containing a *pRS414-GFP-ATG8* plasmid were grown in synthetic glucose medium lacking tryptophan (SC—TRP) to OD_600_ 1.0, then shifted to nitrogen deficient medium (SD-N) for 24 hr. SD-N medium contains YNB, glucose and water.

### Ubiquitination assay

Cells containing *P*_*GAL*_*Flag-Htt103QP-GFP* with either p1217 empty vector or *P*_*GAL*_*HA-Ub* plasmid were grown in raffinose containing synthetic medium without tryptophan (SC—TRP) to OD_600_ = 0.2. Galactose was then added to the medium for 4 hr to induce expression of HA-Ub and Flag-Htt103QP-GFP. Cells were then harvested by centrifugation at 10,000 rpm for 10 min at 4°C. Cells were washed once with water and then resuspended in RIPA buffer (50mM Tris-HCl pH 7.5, 150mM NaCl, 5mM EDTA, 0.05% Tween-20) along with azide and protease inhibitor cocktail (EMD Millipore Corp., Billerica, MA). Samples were then frozen by slowly dripping them into liquid nitrogen to form pellets. Pellets were crushed using a freezer mill. Once crushed, samples were allowed to thaw on ice in the presence of 20mM deubiquitinase inhibitor NEM (Sigma Aldrich). Then, samples were centrifuged at 4,000 rpm for 20 min at 4°C. Supernatant was collected and centrifuged again at 20,000 *g* for 20 min at 4°C. Input samples were saved, then M2 Flag agarose beads (Sigma Aldrich) were used to immunoprecipitate Flag-Htt103QP-GFP. The beads were washed 3 times and resuspended in 1× SDS loading buffer and boiled for 5 min. Western blotting was performed using anti-Flag, anti-HA and anti-Pgk1 antibodies.

### Sedimentation assay

The protocol was described previously with minor adaptation for our experiments [46]. Cells were grown in YEP (yeast extract peptone) medium containing 2% raffinose. Once cells were in mid-log phase, galactose was added to 2% and the cells were incubated at 30°C for 2 hrs. Five mL of cells were harvested and resuspended in 100μL lysis buffer (100mM Tris-HCl pH 7.5, 200mM NaCl, 1mM EDTA, 1mM DTT, 5% glycerol and 0.1% Nonidet P40) plus PMSF with 100μL 0.5mm acid-washed glass beads. Cells were lysed using a beads beater at 4°C. Cell debris were cleared by centrifuging lysates at 700*g* for 1 min at 4°C. We removed 50μL lysate, representing total lysate, and added to 50μL SUMEB (8M Urea, 1% SDS, 10mM MOPS pH 6.8, 10mM EDTA, 1mM PMSF, 0.01% Bromophenol Blue). The remaining lysate (100μL) was centrifuged at 12,800 *g* for 15 min at 4°C. We added 100μL supernatant, representing the soluble fraction, to 100μL SUMEB. The pellet representing the insoluble fraction was resuspended in 100μL lysis buffer and 100μL SUMEB. All samples were incubated at 65°C for 10 minutes and then centrifuged for 5 minutes at 12,800*g*. Proteins were resolved on SDS-PAGE gels, transferred to nitrocellulose membrane and immunoblotted with anti-Flag (Sigma) and anti-Pgk1 (Molecular Probes) antibodies.

### SDS agarose gel electrophoresis (SDS-AGE)

We adapted the protocol from previous work with some modification [31]. Briefly, yeast cells were grown in 10 mL non-inducible raffinose medium until mid-log phase, then galactose was added to 2% for 3 hrs. 1mL of cells was harvested and resuspended in RIPA (0.05% Tween, 5mM EDTA, 50mM Tris-HCl pH 7.5, 150mM NaCl) buffer plus protease inhibitor cocktail (Millipore Calbiochem) and PMSF. Cells were broken using a beads beater. Cell debris were cleared by centrifuging at 300 *g* for 3 min. Sample buffer (150mM Tris, pH 6.8, 33% glycerol, 1.2% SDS, bromophenol blue) was added at equal volume to lysate and samples were incubated at 37°C for 15 min. Samples were then loaded on a 1% agarose gel with running buffer (25mM Tris-HCl, 192mM glycine, and 0.1% SDS). Gels were run at 125V until blue dye reached the bottom. Gel was then semi-dry transferred onto a nitrocellulose membrane for 30 min at constant 0.09 Amps at 4°C. The membrane was probed with anti-Flag antibody (Sigma).

### Statistical analysis

Statistical analysis and significance was performed using student T-test. All error bars shown in this manuscript represent standard error of the mean (SEM). ImageJ was used for quantitation of Western blot images.

## Supporting information

S1 Table Yeast strains used in this study. (DOCX)

S1 Fig Htt103QP inclusion body formation and degradation in chaperone mutants. (A) Fluorescent images of Htt103QP-GFP in WT, *ssa1*Δ, *ssa2*Δ, *ssa3*Δ, and *ssa4*Δ cells after 16 hr induction in galactose medium. Cells were quantified for the number of aggregates in each cell: 1, 2 or > 2 (n = 100 cells). The results are the average of three independent experiments. Scale bar = 5μm. (B) Htt103QP degradation in cytosolic Hsp70 mutants. WT, *ssa1*Δ, *ssa2*Δ, *ssa3*Δ, and *ssa4*Δ cells with *P*_*GAL*_*Flag-Htt103QP-GFP* were grown in non-inducible raffinose containing medium to mid-log phase. Galactose was then added for 1 hr to induce Htt103QP overexpression. Glucose was subsequently added to shut off expression. Cells were collected at indicated time points to determine Htt103QP protein levels. Pgk1: loading control. (C) Fluorescent images of Htt103QP-GFP in WT, *hsp42*Δ, *hsp82*Δ, *hsc82*Δ, *sti1*Δ, and *ssb1*Δ cells after 16 hr induction. Cells were quantified for the number of aggregates in each cell: 1, 2 or > 2 (n = 100 cells). The results are the average of three independent experiments. Scale bar = 5μm. (D). Htt103QP degradation in WT, *hsp42*Δ, *hsp82*Δ, *hsc82*Δ, *sti1*Δ, and *ssb1*Δ cells. Same protocol was used as in (B). All microscopy in this figure was performed on the EVOS microscope.
(TIFF)

S2 Fig Hsp104-GFP localization in WT yeast cells. WT cells containing *HSP104-GFP* were grown in either YPD, YEP+Galactose (YEPG), or YEP+Raffinose (YEPR) for at 30°C for 3 hrs. Cells were then collected, fixed with 70% ethanol, stained with DAPI, and visualized using microscopy. DAPI staining was used to mark the nucleus. Representative GFP and DAPI images are shown. Scale bar = 5μm.
(TIFF)

S3 Fig Proteasome-dependent Htt103QP degradation in HSP mutants. (A) Proteasome inhibitor MG132 blocks Htt103QP degradation after short-time induction. WT cells growing at 30°C in non-inducible (YEP + raffinose) medium containing 0.1% L-proline were treated with 0.003% SDS for 3 hr. Then, either DMSO or 75μM MG132 was added to the cultures for 30 min. Galactose was then added to induce Htt103QP overexpression for 1 hr. Finally, glucose was added to shut off Htt103QP expression. Cells were collected when in galactose (Gal) and also at 90 and 180 min after glucose addition. The Htt103QP protein levels were detected using anti-Flag antibody. Pgk1: loading control. The quantitative degradation kinetics expressed as percent remaining is shown in the right panel. (B) Htt103QP degradation in cells lacking autophagy genes after short-time induction. WT, *atg8*Δ, and *atg7*Δ were grown in non-inducible YEP + raffinose medium to mid log phase, then galactose was added for 1 hr to induce Htt103QP overexpression. Glucose was then added to shut off Htt103QP expression. Samples were collected after 1 hr galactose induction and after glucose addition for 90 and 180 min. The Htt103QP protein levels were detected using anti-Flag antibody. Pgk1: loading control. (C) Htt103QP degradation in WT and *sse1*Δ mutant. Same protocol was used as in (B). The quantitative degradation kinetics expressed at percent remaining is shown in the right panel. (D) Htt103QP degradation in WT, *sse2*Δ, *snl1*Δ *and fes1*Δ cells. Same protocol was used as described in (B). The quantitative degradation kinetics expressed at percent remaining is shown in the right panel. (E) Htt103QP degradation in WT and *ydj1-151* cells. Cells were grown at 25°C in non-inducible YEP + raffinose medium to mid log phase, then galactose was added to induce Htt103QP overexpression for 50 min. Cells were then shifted to 37°C for 10 min before glucose was added. Samples were taken after 1 hr galactose induction (Gal) and after glucose addition for 90 and 180 min. The quantitative Htt103QP degradation is shown in the right panel.
(TIFF)

S4 Fig The solubility and ubiquitination of Htt103QP in WT and *sse1* mutant cells. (A) Htt103QP sedimentation assay in WT and *sse1*Δ cells. Cells were grown in non-inducible raffinose medium at 30°C to mid-log phase, then galactose was added to 2% for 2 hr. Cells were lysed with beads beater and Htt103QP was fractionated into soluble (S) and insoluble (I) fractions by centrifugation. The preparation of the T (total), S and I fractions was described in the Materials and Methods section. Anti-Flag antibody was used to detect Htt103QP, and anti-Pgk1 antibody was used to determine the distribution of Pgk1 in each fraction. (B) Htt103QP ubiquitination in WT and *sse1*Δ cells. WT and *sse1*Δ cells carrying *P*_*GAL*_*Flag-Htt103QP-GFP* and *P*_*GAL*_*HA-Ub* or *P*_*GAL*_*-HA* were grown in raffinose containing medium to early log phase. Galactose was added to induce Htt103QP and Ub overexpression for 4 hr. Flag-Htt103QP-GFP was immunoprecipitated (IP) using anti-Flag M2 agarose beads. Anti-Flag antibody was used to detect Htt103QP protein level. Anti-HA antibody was used to detect Ub protein level. Pgk1: loading control.
(TIFF)

## References

[1] Soto C. Unfolding the role of protein misfolding in neurodegenerative diseases. Nat Rev Neurosci. 2003;4(1):49–60. doi: 10.1038/nrn1007 . 12511861 10.1038/nrn1007

[2] Knowles TP, Vendruscolo M, Dobson CM. The amyloid state and its association with protein misfolding diseases. Nat Rev Mol Cell Biol. 2014;15(6):384–96. doi: 10.1038/nrm3810 . 24854788 10.1038/nrm3810

[3] de Oliveira GA, Rangel LP, Costa DC, Silva JL. Misfolding, Aggregation, and Disordered Segments in c-Abl and p53 in Human Cancer. Front Oncol. 2015;5:97 doi: 10.3389/fonc.2015.00097 25973395 10.3389/fonc.2015.00097 PMC4413674

[4] Mukherjee A, Morales-Scheihing D, Butler PC, Soto C. Type 2 diabetes as a protein misfolding disease. Trends Mol Med. 2015;21(7):439–49. doi: 10.1016/j.molmed.2015.04.005 25998900 10.1016/j.molmed.2015.04.005 PMC4492843

[5] Hartl FU, Bracher A, Hayer-Hartl M. Molecular chaperones in protein folding and proteostasis. Nature. 2011;475(7356):324–32. doi: 10.1038/nature10317 . 21776078 10.1038/nature10317

[6] Mayer MP. Hsp70 chaperone dynamics and molecular mechanism. Trends Biochem Sci. 2013;38(10):507–14. doi: 10.1016/j.tibs.2013.08.001 . 24012426 10.1016/j.tibs.2013.08.001

[7] Kityk R, Kopp J, Sinning I, Mayer MP. Structure and dynamics of the ATP-bound open conformation of Hsp70 chaperones. Mol Cell. 2012;48(6):863–74. doi: 10.1016/j.molcel.2012.09.023 . 23123194 10.1016/j.molcel.2012.09.023

[8] Ghaemmaghami S, Huh WK, Bower K, Howson RW, Belle A, Dephoure N, et al Global analysis of protein expression in yeast. Nature. 2003;425(6959):737–41. doi: 10.1038/nature02046 . 14562106 10.1038/nature02046

[9] Abrams JL, Verghese J, Gibney PA, Morano KA. Hierarchical functional specificity of cytosolic heat shock protein 70 (Hsp70) nucleotide exchange factors in yeast. J Biol Chem. 2014;289(19):13155–67. doi: 10.1074/jbc.M113.530014 24671421 10.1074/jbc.M113.530014 PMC4036327

[10] Misselwitz B, Staeck O, Rapoport TA. J proteins catalytically activate Hsp70 molecules to trap a wide range of peptide sequences. Mol Cell. 1998;2(5):593–603. . 9844632 10.1016/s1097-2765(00)80158-6

[11] Cyr DM, Ramos CH. Specification of Hsp70 function by Type I and Type II Hsp40. Subcell Biochem. 2015;78:91–102. doi: 10.1007/978-3-319-11731-7_4 . 25487017 10.1007/978-3-319-11731-7_4

[12] Nillegoda NB, Bukau B. Metazoan Hsp70-based protein disaggregases: emergence and mechanisms. Front Mol Biosci. 2015;2:57 doi: 10.3389/fmolb.2015.00057 26501065 10.3389/fmolb.2015.00057 PMC4598581

[13] Kaimal JM, Kandasamy G, Gasser F, Andreasson C. Coordinated Hsp110 and Hsp104 activities power protein disaggregation in Saccharomyces cerevisiae. Mol Cell Biol. 2017 doi: 10.1128/MCB.00027-17 . 28289075 10.1128/MCB.00027-17 PMC5440654

[14] Torrente MP, Shorter J. The metazoan protein disaggregase and amyloid depolymerase system: Hsp110, Hsp70, Hsp40, and small heat shock proteins. Prion. 2013;7(6):457–63. doi: 10.4161/pri.27531 24401655 10.4161/pri.27531 PMC4201613

[15] Rampelt H, Kirstein-Miles J, Nillegoda NB, Chi K, Scholz SR, Morimoto RI, et al Metazoan Hsp70 machines use Hsp110 to power protein disaggregation. EMBO J. 2012;31(21):4221–35. doi: 10.1038/emboj.2012.264 22990239 10.1038/emboj.2012.264 PMC3492728

[16] Park SH, Kukushkin Y, Gupta R, Chen T, Konagai A, Hipp MS, et al PolyQ proteins interfere with nuclear degradation of cytosolic proteins by sequestering the Sis1p chaperone. Cell. 2013;154(1):134–45. doi: 10.1016/j.cell.2013.06.003 . 23791384 10.1016/j.cell.2013.06.003

[17] Olzscha H, Schermann SM, Woerner AC, Pinkert S, Hecht MH, Tartaglia GG, et al Amyloid-like aggregates sequester numerous metastable proteins with essential cellular functions. Cell. 2011;144(1):67–78. doi: 10.1016/j.cell.2010.11.050 . 21215370 10.1016/j.cell.2010.11.050

[18] Keller JN, Hanni KB, Markesbery WR. Impaired proteasome function in Alzheimer’s disease. J Neurochem. 2000;75(1):436–9. . 10854289 10.1046/j.1471-4159.2000.0750436.x

[19] Hwang JS, Hwang JS, Chang I, Kim S. Age-associated decrease in proteasome content and activities in human dermal fibroblasts: restoration of normal level of proteasome subunits reduces aging markers in fibroblasts from elderly persons. J Gerontol A Biol Sci Med Sci. 2007;62(5):490–9. . 17522352 10.1093/gerona/62.5.490

[20] Tydlacka S, Wang CE, Wang X, Li S, Li XJ. Differential activities of the ubiquitin-proteasome system in neurons versus glia may account for the preferential accumulation of misfolded proteins in neurons. J Neurosci. 2008;28(49):13285–95. doi: 10.1523/JNEUROSCI.4393-08.2008 19052220 10.1523/JNEUROSCI.4393-08.2008 PMC2662777

[21] Low P. The role of ubiquitin-proteasome system in ageing. Gen Comp Endocrinol. 2011;172(1):39–43. doi: 10.1016/j.ygcen.2011.02.005 . 21324320 10.1016/j.ygcen.2011.02.005

[22] Ciechanover A, Kwon YT. Protein Quality Control by Molecular Chaperones in Neurodegeneration. Front Neurosci. 2017;11:185 doi: 10.3389/fnins.2017.00185 28428740 10.3389/fnins.2017.00185 PMC5382173

[23] Duennwald ML, Jagadish S, Muchowski PJ, Lindquist S. Flanking sequences profoundly alter polyglutamine toxicity in yeast. Proc Natl Acad Sci U S A. 2006;103(29):11045–50. Epub 2006/07/13. doi: 10.1073/pnas.0604547103 . 16832050 10.1073/pnas.0604547103 PMC1544171

[24] Krobitsch S, Lindquist S. Aggregation of huntingtin in yeast varies with the length of the polyglutamine expansion and the expression of chaperone proteins. Proc Natl Acad Sci U S A. 2000;97(4):1589–94. 10677504 10.1073/pnas.97.4.1589 PMC26479

[25] Wang Y, Meriin AB, Zaarur N, Romanova NV, Chernoff YO, Costello CE, et al Abnormal proteins can form aggresome in yeast: aggresome-targeting signals and components of the machinery. FASEB J. 2009;23(2):451–63. doi: 10.1096/fj.08-117614 18854435 10.1096/fj.08-117614 PMC2630789

[26] Chuang KH, Liang F, Higgins R, Wang Y. Ubiquilin/Dsk2 promotes inclusion body formation and vacuole (lysosome)-mediated disposal of mutated huntingtin. Mol Biol Cell. 2016;27(13):2025–36. doi: 10.1091/mbc.E16-01-0026 27170182 10.1091/mbc.E16-01-0026 PMC4927277

[27] Yang J, Hao X, Cao X, Liu B, Nystrom T. Spatial sequestration and detoxification of Huntingtin by the ribosome quality control complex. Elife. 2016;5 doi: 10.7554/eLife.11792 27033550 10.7554/eLife.11792 PMC4868537

[28] Gong B, Kielar C, Morton AJ. Temporal separation of aggregation and ubiquitination during early inclusion formation in transgenic mice carrying the Huntington’s disease mutation. PLoS One. 2012;7(7):e41450 doi: 10.1371/journal.pone.0041450 22848498 10.1371/journal.pone.0041450 PMC3404089

[29] Shaner L, Wegele H, Buchner J, Morano KA. The yeast Hsp110 Sse1 functionally interacts with the Hsp70 chaperones Ssa and Ssb. J Biol Chem. 2005;280(50):41262–9. doi: 10.1074/jbc.M503614200 . 16221677 10.1074/jbc.M503614200

[30] Dragovic Z, Broadley SA, Shomura Y, Bracher A, Hartl FU. Molecular chaperones of the Hsp110 family act as nucleotide exchange factors of Hsp70s. EMBO J. 2006;25(11):2519–28. doi: 10.1038/sj.emboj.7601138 16688212 10.1038/sj.emboj.7601138 PMC1478182

[31] Shen K, Calamini B, Fauerbach JA, Ma B, Shahmoradian SH, Serrano Lachapel IL, et al Control of the structural landscape and neuronal proteotoxicity of mutant Huntingtin by domains flanking the polyQ tract. Elife. 2016;5 doi: 10.7554/eLife.18065 27751235 10.7554/eLife.18065 PMC5135392

[32] Morano KA. New tricks for an old dog: the evolving world of Hsp70. Ann N Y Acad Sci. 2007;1113:1–14. doi: 10.1196/annals.1391.018 . 17513460 10.1196/annals.1391.018

[33] Bracher A, Verghese J. The nucleotide exchange factors of Hsp70 molecular chaperones. Front Mol Biosci. 2015;2:10 doi: 10.3389/fmolb.2015.00010 26913285 10.3389/fmolb.2015.00010 PMC4753570

[34] Gong Y, Kakihara Y, Krogan N, Greenblatt J, Emili A, Zhang Z, et al An atlas of chaperone-protein interactions in Saccharomyces cerevisiae: implications to protein folding pathways in the cell. Mol Syst Biol. 2009;5:275 doi: 10.1038/msb.2009.26 19536198 10.1038/msb.2009.26 PMC2710862

[35] Needham PG, Patel HJ, Chiosis G, Thibodeau PH, Brodsky JL. Mutations in the Yeast Hsp70, Ssa1, at P417 Alter ATP Cycling, Interdomain Coupling, and Specific Chaperone Functions. J Mol Biol. 2015;427(18):2948–65. doi: 10.1016/j.jmb.2015.04.010 25913688 10.1016/j.jmb.2015.04.010 PMC4569534

[36] Kabani M, Beckerich JM, Brodsky JL. Nucleotide exchange factor for the yeast Hsp70 molecular chaperone Ssa1p. Mol Cell Biol. 2002;22(13):4677–89. doi: 10.1128/MCB.22.13.4677-4689.2002 12052876 10.1128/MCB.22.13.4677-4689.2002 PMC133915

[37] Sondheimer N, Lopez N, Craig EA, Lindquist S. The role of Sis1 in the maintenance of the [RNQ+] prion. EMBO J. 2001;20(10):2435–42. doi: 10.1093/emboj/20.10.2435 11350932 10.1093/emboj/20.10.2435 PMC125465

[38] Meriin AB, Zhang X, He X, Newnam GP, Chernoff YO, Sherman MY. Huntington toxicity in yeast model depends on polyglutamine aggregation mediated by a prion-like protein Rnq1. J Cell Biol. 2002;157(6):997–1004. doi: 10.1083/jcb.200112104 12058016 10.1083/jcb.200112104 PMC2174031

[39] Glover JR, Lindquist S. Hsp104, Hsp70, and Hsp40: a novel chaperone system that rescues previously aggregated proteins. Cell. 1998;94(1):73–82. . 9674429 10.1016/s0092-8674(00)81223-4

[40] Parsell DA, Kowal AS, Singer MA, Lindquist S. Protein disaggregation mediated by heat-shock protein Hsp104. Nature. 1994;372(6505):475–8. doi: 10.1038/372475a0 . 7984243 10.1038/372475a0

[41] O′Driscoll J, Clare D, Saibil H. Prion aggregate structure in yeast cells is determined by the Hsp104-Hsp110 disaggregase machinery. J Cell Biol. 2015;211(1):145–58. doi: 10.1083/jcb.201505104 26438827 10.1083/jcb.201505104 PMC4602031

[42] Tkach JM, Glover JR. Nucleocytoplasmic trafficking of the molecular chaperone Hsp104 in unstressed and heat-shocked cells. Traffic. 2008;9(1):39–56. doi: 10.1111/j.1600-0854.2007.00666.x . 17973656 10.1111/j.1600-0854.2007.00666.x

[43] Kempf C, Lengeler K, Wendland J. Differential stress response of Saccharomyces hybrids revealed by monitoring Hsp104 aggregation and disaggregation. Microbiol Res. 2017;200:53–63. doi: 10.1016/j.micres.2017.03.009 . 28527764 10.1016/j.micres.2017.03.009

[44] Li XJ, Li H, Li S. Clearance of mutant huntingtin. Autophagy. 2010;6(5):663–4. . 20519964 10.4161/auto.6.5.12336 PMC5822432

[45] Shiber A, Breuer W, Brandeis M, Ravid T. Ubiquitin conjugation triggers misfolded protein sequestration into quality control foci when Hsp70 chaperone levels are limiting. Mol Biol Cell. 2013;24(13):2076–87. doi: 10.1091/mbc.E13-01-0010 23637465 10.1091/mbc.E13-01-0010 PMC3694792

[46] Gallagher PS, Clowes Candadai SV, Gardner RG. The requirement for Cdc48/p97 in nuclear protein quality control degradation depends on the substrate and correlates with substrate insolubility. J Cell Sci. 2014;127(Pt 9):1980–91. doi: 10.1242/jcs.141838 24569878 10.1242/jcs.141838 PMC4004975

[47] Varshavsky A. The ubiquitin system, an immense realm. Annual review of biochemistry. 2012;81:167–76. doi: 10.1146/annurev-biochem-051910-094049 . 22663079 10.1146/annurev-biochem-051910-094049

[48] McClellan AJ, Scott MD, Frydman J. Folding and quality control of the VHL tumor suppressor proceed through distinct chaperone pathways. Cell. 2005;121(5):739–48. doi: 10.1016/j.cell.2005.03.024 . 15935760 10.1016/j.cell.2005.03.024

[49] Heck JW, Cheung SK, Hampton RY. Cytoplasmic protein quality control degradation mediated by parallel actions of the E3 ubiquitin ligases Ubr1 and San1. Proc Natl Acad Sci U S A. 2010;107(3):1106–11. doi: 10.1073/pnas.0910591107 20080635 10.1073/pnas.0910591107 PMC2824284

[50] Prasad R, Kawaguchi S, Ng DT. A nucleus-based quality control mechanism for cytosolic proteins. Mol Biol Cell. 2010;21(13):2117–27. doi: 10.1091/mbc.E10-02-0111 20462951 10.1091/mbc.E10-02-0111 PMC2893977

[51] Gowda NK, Kandasamy G, Froehlich MS, Dohmen RJ, Andreasson C. Hsp70 nucleotide exchange factor Fes1 is essential for ubiquitin-dependent degradation of misfolded cytosolic proteins. Proc Natl Acad Sci U S A. 2013;110(15):5975–80. doi: 10.1073/pnas.1216778110 23530227 10.1073/pnas.1216778110 PMC3625341

[52] Gowda NK, Kaimal JM, Masser AE, Kang W, Friedlander MR, Andreasson C. Cytosolic splice isoform of Hsp70 nucleotide exchange factor Fes1 is required for the degradation of misfolded proteins in yeast. Mol Biol Cell. 2016;27(8):1210–9. doi: 10.1091/mbc.E15-10-0697 26912797 10.1091/mbc.E15-10-0697 PMC4831876

[53] Brehme M, Voisine C, Rolland T, Wachi S, Soper JH, Zhu Y, et al A chaperome subnetwork safeguards proteostasis in aging and neurodegenerative disease. Cell Rep. 2014;9(3):1135–50. doi: 10.1016/j.celrep.2014.09.042 25437566 10.1016/j.celrep.2014.09.042 PMC4255334

[54] Wolfe KJ, Ren HY, Trepte P, Cyr DM. The Hsp70/90 cochaperone, Sti1, suppresses proteotoxicity by regulating spatial quality control of amyloid-like proteins. Mol Biol Cell. 2013;24(23):3588–602. doi: 10.1091/mbc.E13-06-0315 24109600 10.1091/mbc.E13-06-0315 PMC3842988

[55] Bersuker K, Hipp MS, Calamini B, Morimoto RI, Kopito RR. Heat shock response activation exacerbates inclusion body formation in a cellular model of Huntington disease. J Biol Chem. 2013;288(33):23633–8. doi: 10.1074/jbc.C113.481945 23839939 10.1074/jbc.C113.481945 PMC3745309

[56] Shahmoradian SH, Galaz-Montoya JG, Schmid MF, Cong Y, Ma B, Spiess C, et al TRiC’s tricks inhibit huntingtin aggregation. Elife. 2013;2:e00710 doi: 10.7554/eLife.00710 23853712 10.7554/eLife.00710 PMC3707056

[57] Qi L, Zhang XD, Wu JC, Lin F, Wang J, DiFiglia M, et al The role of chaperone-mediated autophagy in huntingtin degradation. PLoS One. 2012;7(10):e46834 doi: 10.1371/journal.pone.0046834 23071649 10.1371/journal.pone.0046834 PMC3469570

[58] Zaffagnini G, Martens S. Mechanisms of Selective Autophagy. J Mol Biol. 2016;428(9 Pt A):1714–24. doi: 10.1016/j.jmb.2016.02.004 26876603 10.1016/j.jmb.2016.02.004 PMC4871809

[59] Nair U, Thumm M, Klionsky DJ, Krick R. GFP-Atg8 protease protection as a tool to monitor autophagosome biogenesis. Autophagy. 2011;7(12):1546–50. doi: 10.4161/auto.7.12.18424 22108003 10.4161/auto.7.12.18424 PMC3327617

[60] Gidalevitz T, Ben-Zvi A, Ho KH, Brignull HR, Morimoto RI. Progressive disruption of cellular protein folding in models of polyglutamine diseases. Science. 2006;311(5766):1471–4. doi: 10.1126/science.1124514 . 16469881 10.1126/science.1124514

[61] Douglas PM, Dillin A. Protein homeostasis and aging in neurodegeneration. J Cell Biol. 2010;190(5):719–29. doi: 10.1083/jcb.201005144 20819932 10.1083/jcb.201005144 PMC2935559

[62] Bhat KP, Yan S, Wang CE, Li S, Li XJ. Differential ubiquitination and degradation of huntingtin fragments modulated by ubiquitin-protein ligase E3A. Proc Natl Acad Sci U S A. 2014;111(15):5706–11. doi: 10.1073/pnas.1402215111 24706802 10.1073/pnas.1402215111 PMC3992696

[63] Lu K, Psakhye I, Jentsch S. Autophagic clearance of polyQ proteins mediated by ubiquitin-Atg8 adaptors of the conserved CUET protein family. Cell. 2014;158(3):549–63. doi: 10.1016/j.cell.2014.05.048 . 25042851 10.1016/j.cell.2014.05.048

[64] Kaganovich D, Kopito R, Frydman J. Misfolded proteins partition between two distinct quality control compartments. Nature. 2008;454(7208):1088–95. Epub 2008/08/30. doi: 10.1038/nature07195 18756251 10.1038/nature07195 PMC2746971

[65] Bauerlein FJB, Saha I, Mishra A, Kalemanov M, Martinez-Sanchez A, Klein R, et al In Situ Architecture and Cellular Interactions of PolyQ Inclusions. Cell. 2017;171(1):179–87 e10. doi: 10.1016/j.cell.2017.08.009 . 28890085 10.1016/j.cell.2017.08.009

[66] Xu Z, Graham K, Foote M, Liang F, Rizkallah R, Hurt M, et al 14-3-3 protein targets misfolded chaperone-associated proteins to aggresomes. J Cell Sci. 2013;126(Pt 18):4173–86. doi: 10.1242/jcs.126102 23843611 10.1242/jcs.126102 PMC3772389

[67] Leitman J, Ulrich Hartl F, Lederkremer GZ. Soluble forms of polyQ-expanded huntingtin rather than large aggregates cause endoplasmic reticulum stress. Nat Commun. 2013;4:2753 doi: 10.1038/ncomms3753 . 24217578 10.1038/ncomms3753

[68] Duennwald ML. Polyglutamine misfolding in yeast: toxic and protective aggregation. Prion. 2011;5(4):285–90. doi: 10.4161/pri.18071 22052348 10.4161/pri.5.4.18071 PMC4012402

[69] Nagy M, Fenton WA, Li D, Furtak K, Horwich AL. Extended survival of misfolded G85R SOD1-linked ALS mice by transgenic expression of chaperone Hsp110. Proc Natl Acad Sci U S A. 2016;113(19):5424–8. doi: 10.1073/pnas.1604885113 27114530 10.1073/pnas.1604885113 PMC4868459

[70] Eroglu B, Moskophidis D, Mivechi NF. Loss of Hsp110 leads to age-dependent tau hyperphosphorylation and early accumulation of insoluble amyloid beta. Mol Cell Biol. 2010;30(19):4626–43. doi: 10.1128/MCB.01493-09 20679486 10.1128/MCB.01493-09 PMC2950521

[71] Longtine MS, McKenzie A 3rd, Demarini DJ, Shah NG, Wach A, Brachat A, et al Additional modules for versatile and economical PCR-based gene deletion and modification in Saccharomyces cerevisiae. Yeast. 1998;14(10):953–61. doi: 10.1002/(SICI)1097-0061(199807)14:10<953::AID-YEA293>3.0.CO;2-U . 9717241 10.1002/(SICI)1097-0061(199807)14:10<953::AID-YEA293>3.0.CO;2-U

[72] Abeliovich H, Zhang C, Dunn WA Jr., Shokat KM, Klionsky DJ. Chemical genetic analysis of Apg1 reveals a non-kinase role in the induction of autophagy. Mol Biol Cell. 2003;14(2):477–90. doi: 10.1091/mbc.E02-07-0413 12589048 10.1091/mbc.E02-07-0413 PMC149986

[73] Lim KL, Chew KC, Tan JM, Wang C, Chung KK, Zhang Y, et al Parkin mediates nonclassical, proteasomal-independent ubiquitination of synphilin-1: implications for Lewy body formation. J Neurosci. 2005;25(8):2002–9. doi: 10.1523/JNEUROSCI.4474-04.2005 . 15728840 10.1523/JNEUROSCI.4474-04.2005 PMC6726069

