# Supplementary material for: The absence of specific yeast heat-shock proteins leads to abnormal aggregation and compromised autophagic clearance of mutant Huntingtin proteins
Source: PLoS One. 2018 Jan 18;13(1):e0191490. doi: 10.1371/journal.pone.0191490 (PMC5773196; doi:10.1371/journal.pone.0191490)
Supplement: S1 Table — (DOCX) [file pone.0191490.s001.docx]

**S1 Table. Yeast strains used in this study.**

| **Strains** | **Relevant genotypes** | **Reference** |
| --- | --- | --- |
| Y300 | Mata *ura3-1, his3-11,15 leu2-3,112 trp1-1, ade2-1, can1-100* | Lab Stock |
| 3419-1-1 | Mata Y300 with *P_GAL_-Flag-Htt103QP-GFP::URA3* | This study |
| 3361-1-4 | Matα *P_GAL_Flag-Htt103QP-GFP::URA3, sse1::KanMX6* | This study |
| 3361-3-3 | Mata *P_GAL_Flag-Htt103QP-GFP::URA3, sse1::KanMX6* | This study |
| 3420-3-3 | Mata *P_GAL_Flag-Htt103QP-GFP::URA3, sse1::sphis5^+^* | This study |
| RH130 | Mata *P_GAL_Flag-Htt103QP-GFP::URA3, fes1::sphis5^+^* | This study |
| 3464-1-2 | Mata *P_GAL_Flag-Htt103QP-GFP::URA3, sse1::KanMX6, fes1::sphis5^+^* | This study |
| 3166-4-3 | Mata *P_GAL_Flag-Htt103QP-GFP::URA3, ydj1-2::HIS3 ydj1-151::LEU2* | This study |
| 3365-1-4 | Mata *P_GAL_Flag-Htt103QP-GFP::URA3, sis1::HIS3, SIS1-HA::LEU2* | This study |
| 3366-2-4 | Mata *P_GAL_Flag-Htt103QP-GFP::URA3, sis1::HIS3, SIS1-85-HA::LEU2* | This study |
| 3426-1-3 | Mata *P_GAL_Flag-Htt103QP-GFP::URA3, atg7::KanMX6* | This study |
| 3420-8-2 | Mata *P_GAL_Flag-Htt103QP-GFP::URA3, atg8::KanMX6* | This study |
| 3272-1-4 | Mata *P_GAL_Flag-Htt103QP-GFP::URA3, ssa1::KanMX6* | This study |
| 3368-2-3 | Mata *P_GAL_Flag-Htt103QP-GFP::URA3, ssa2::KanMX6* | This study |
| 3369-2-4 | Matα *P_GAL_Flag-Htt103QP-GFP::URA3, ssa3::KanMX6* | This study |
| 3371-1-4 | Mata *P_GAL_Flag-Htt103QP-GFP::URA3, ssa4::KanMX6* | This study |
| 3410-2-1 | Mata *P_GAL_Flag-Htt103QP-GFP::URA3, ssb1::KanMX6* | This study |
| 3364-1-2 | Mata *P_GAL_Flag-Htt103QP-GFP::URA3, sti1::KanMX6* | This study |
| 3363-3-2 | Matα *P_GAL_Flag-Htt103QP-GFP::URA3, hsp42::KanMX6* | This study |
| 3286-1-4 | Mata *P_GAL_Flag-Htt103QP-GFP::URA3, hsp82::KanMX6* | This study |
| 3293-1-3 | Mata *P_GAL_Flag-Htt103QP-GFP::URA3, hsc82::KanMX6* | This study |
| 3450-1-2 | Mata *P_GAL_Flag-Htt103QP-GFP::URA3, snl1::KanMX6* | This study |
| 3451-2-3 | Mata *P_GAL_Flag-Htt103QP-GFP::URA3, sse2::KanMX6* | This study |
| RH088-1 | Mata *P_GAL_Flag-Htt103QP-mApple::URA3, pRS414-GFP-ATG8* | This study |
| RH103 | Mata *P_GAL_Flag-Htt103QP-mApple::URA3, pRS414-GFP-ATG8, sse1::sphis5^+^* | This study |
| RH139 | Matα *P_GAL_Flag-Htt103QP-mApple::URA3, pRS414-GFP-ATG8, fes1::sphis5^+^* | This study |
| 3216-1-1 | Mata *P_GAL_Flag-Htt103QP-GFP::URA3, VPH1-mApple::sphis5^+^, pep4::KanMX6* | This study |
| 3276-1-2 | Mata *P_GAL_Flag-Htt103QP-GFP::URA3, VPH1-mApple::sphis5^+^, pep4::KanMX4, sse1::sphis5^+^* | This study |
| 3459-1-1 | Mata *P_GAL_Flag-Htt103QP-GFP::URA3, VPH1-mApple::sphis5^+^, pep4::KanMX6, fes1::sphis5^+^* | This study |
| RH118 | Matα *P_GAL_Flag-Htt103QP-GFP::URA3, VPH1-mApple::sphis5^+^, pep4::KanMX6, ydj1-2::HIS3, ydj1-151::TRP1* | This study |
| FY-12-1 | Mata *P_GAL_Flag-Htt103QP-GFP::URA3, p1217*(empty vector *TRP1*) | This study |
| FY-12-1 | Mata *P_GAL_Flag-Htt103QP-GFP::URA3, P_GAL_HA-Ub* | This study |
| RH095 | Matα *P_GAL_Flag-Htt103QP-GFP::URA3, sse1::sphis5^+^, p1217* (empty vector *TRP1*) | This study |
| RH096 | Matα *P_GAL_Flag-Htt103QP-GFP::URA3, sse1:: sphis5^+^, P_GAL_HA-Ub* | This study |
| RH140 | Matα *SSA1-WT, ssa2-1::LEU2, ssa3-1::TRP1, ssa4-2::LYS2* + pRS416-*P_GAL_Flag-Htt103QP-GFP::URA3,* | This study |
| RH141 | Matα *SSA1-45, ssa2-1::LEU2, ssa3-1::TRP1, ssa4-2::LYS2* + pRS416-*P_GAL_Flag-Htt103QP-GFP::URA3,* | This study |
| YYW315-2 | Mata *HSP104-GFP::TRP1* | This study |
| 3472-6-2 | Mata *HSP104-GFP::TRP1, sse1::sphis5^+^* | This study |
| 3472-1-1 | Mata *P_GAL_Flag-Htt103QP-mApple::URA3, HSP104-GFP::TRP1, sse1::sphis5^+^* | This study |
| 3470-4-3 | Mata *HSP104-GFP::TRP1 fes1:: sphis5^+^* | This study |
| 3470-3-3 | Mata *P_GAL_Flag-Htt103QP-mApple::URA3, HSP104-GFP::TRP1 fes1::sphis5^+^* | This study |
| 3537-1-3 | Mata *HSP104-GFP::TRP1, snl1::KanMX6* | This study |
| 3537-4-1 | Mata *P_GAL_Flag-Htt103QP-mApple::URA3, HSP104-GFP::TRP1, snl1::KanMX6* | This study |
| 3538-1-1 | Mata *HSP104-GFP::TRP1, sse2::KanMX6* | This study |
| 3538-7-2 | Matα *P_GAL_Flag-Htt103QP-mApple::URA3, HSP104-GFP::TRP1, sse2::KanMX6* | This study |
| 3542-1-1 | Mata *HSP104-GFP::TRP1, ydj1-2::HIS3, ydj1-151::LEU2* | This study |
| 3542-2-3 | Mata *P_GAL_Flag-Htt103QP-mApple::URA3, HSP104-GFP::TRP1, ydj1-2::HIS3, ydj1-151::LEU2* | This study |
