# Supplementary figures and images for: The absence of specific yeast heat-shock proteins leads to abnormal aggregation and compromised autophagic clearance of mutant Huntingtin proteins
Source: PLoS One. 2018 Jan 18;13(1):e0191490. doi: 10.1371/journal.pone.0191490 (PMC5773196; doi:10.1371/journal.pone.0191490)

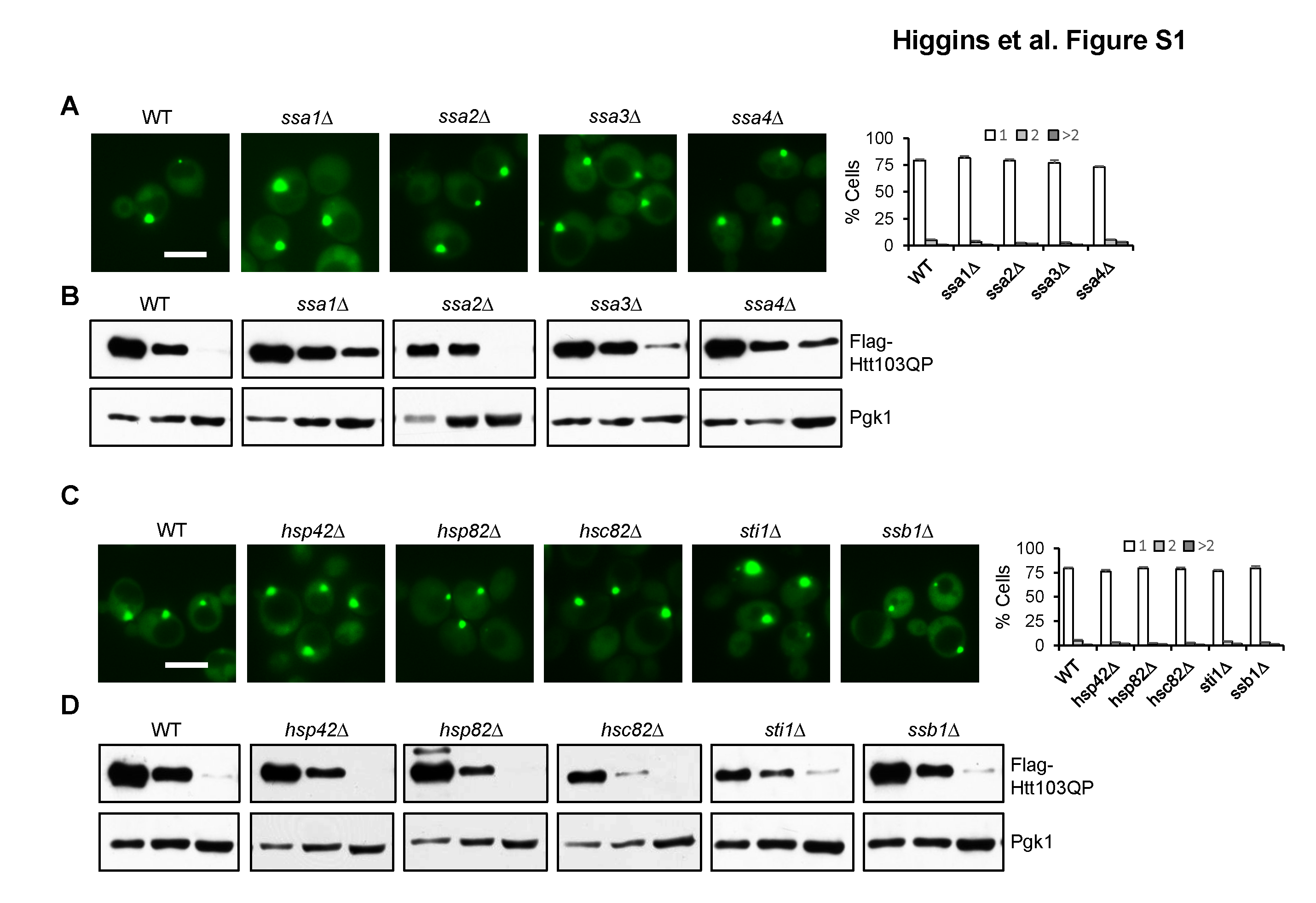

Supplement: S1 Fig — (A) Fluorescent images of Htt103QP-GFP in WT, ssa1Δ, ssa2Δ, ssa3Δ, and ssa4Δ cells after 16 hr induction in galactose medium. Cells were quantified for the number of aggregates in each cell: 1, 2 or > 2 (n = 100 cells). The results are the average of three independent experiments. Scale bar = 5μm. (B) Htt103QP degradation in cytosolic Hsp70 mutants. WT, ssa1Δ, ssa2Δ, ssa3Δ, and ssa4Δ cells with PGALFlag-Htt103QP-GFP were grown in non-inducible raffinose containing medium to mid-log phase. Galactose was then added for 1 hr to induce Htt103QP overexpression. Glucose was subsequently added to shut off expression. Cells were collected at indicated time points to determine Htt103QP protein levels. Pgk1: loading control. (C) Fluorescent images of Htt103QP-GFP in WT, hsp42Δ, hsp82Δ, hsc82Δ, sti1Δ, and ssb1Δ cells after 16 hr induction. Cells were quantified for the number of aggregates in each cell: 1, 2 or > 2 (n = 100 cells). The results are the average of three independent experiments. Scale bar = 5μm. (D). Htt103QP degradation in WT, hsp42Δ, hsp82Δ, hsc82Δ, sti1Δ, and ssb1Δ cells. Same protocol was used as in (B). All microscopy in this figure was performed on the EVOS microscope. (TIFF) [file pone.0191490.s002.tiff]

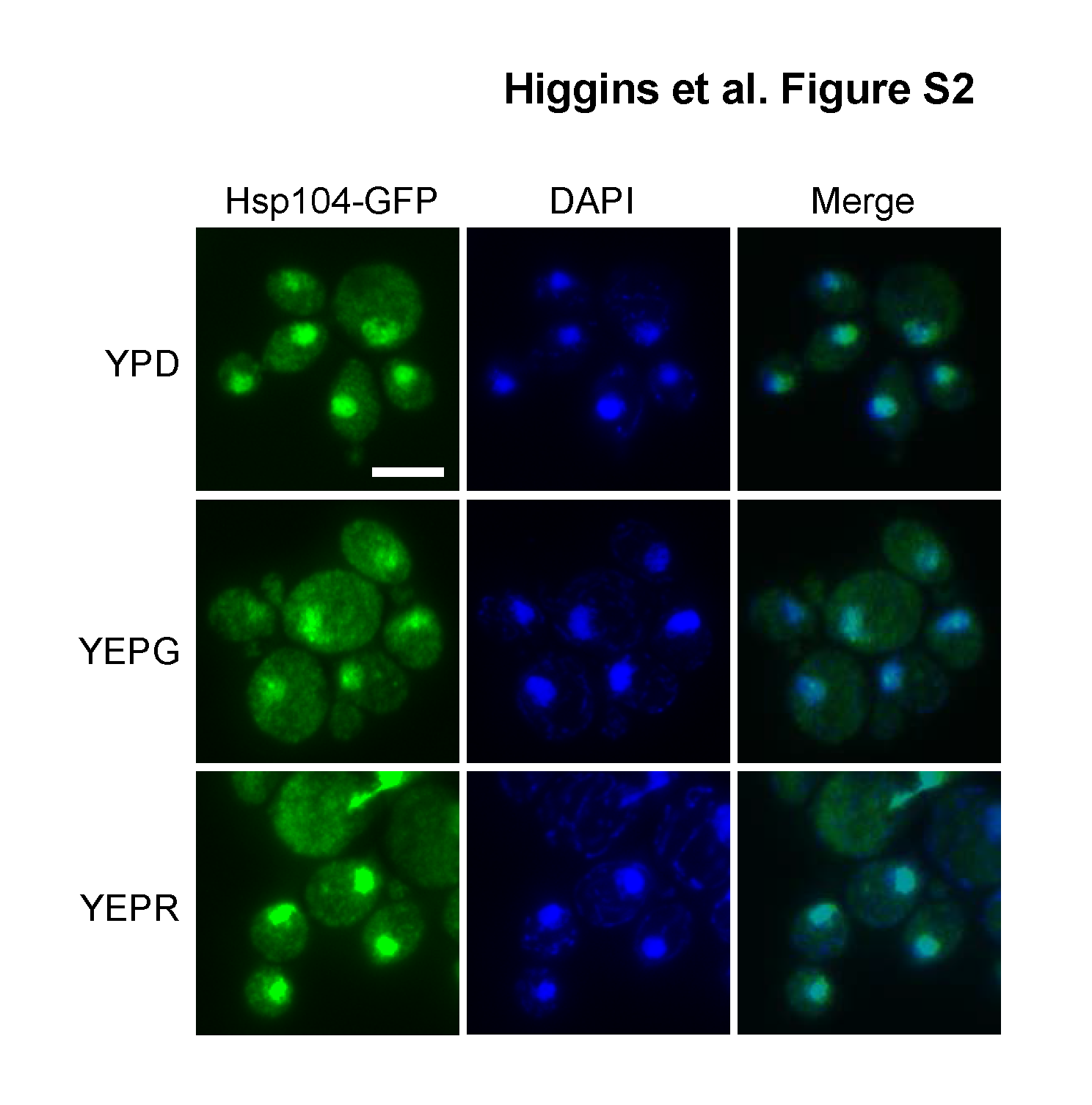

Supplement: S2 Fig — WT cells containing HSP104-GFP were grown in either YPD, YEP+Galactose (YEPG), or YEP+Raffinose (YEPR) for at 30°C for 3 hrs. Cells were then collected, fixed with 70% ethanol, stained with DAPI, and visualized using microscopy. DAPI staining was used to mark the nucleus. Representative GFP and DAPI images are shown. Scale bar = 5μm. (TIFF) [file pone.0191490.s003.tiff]

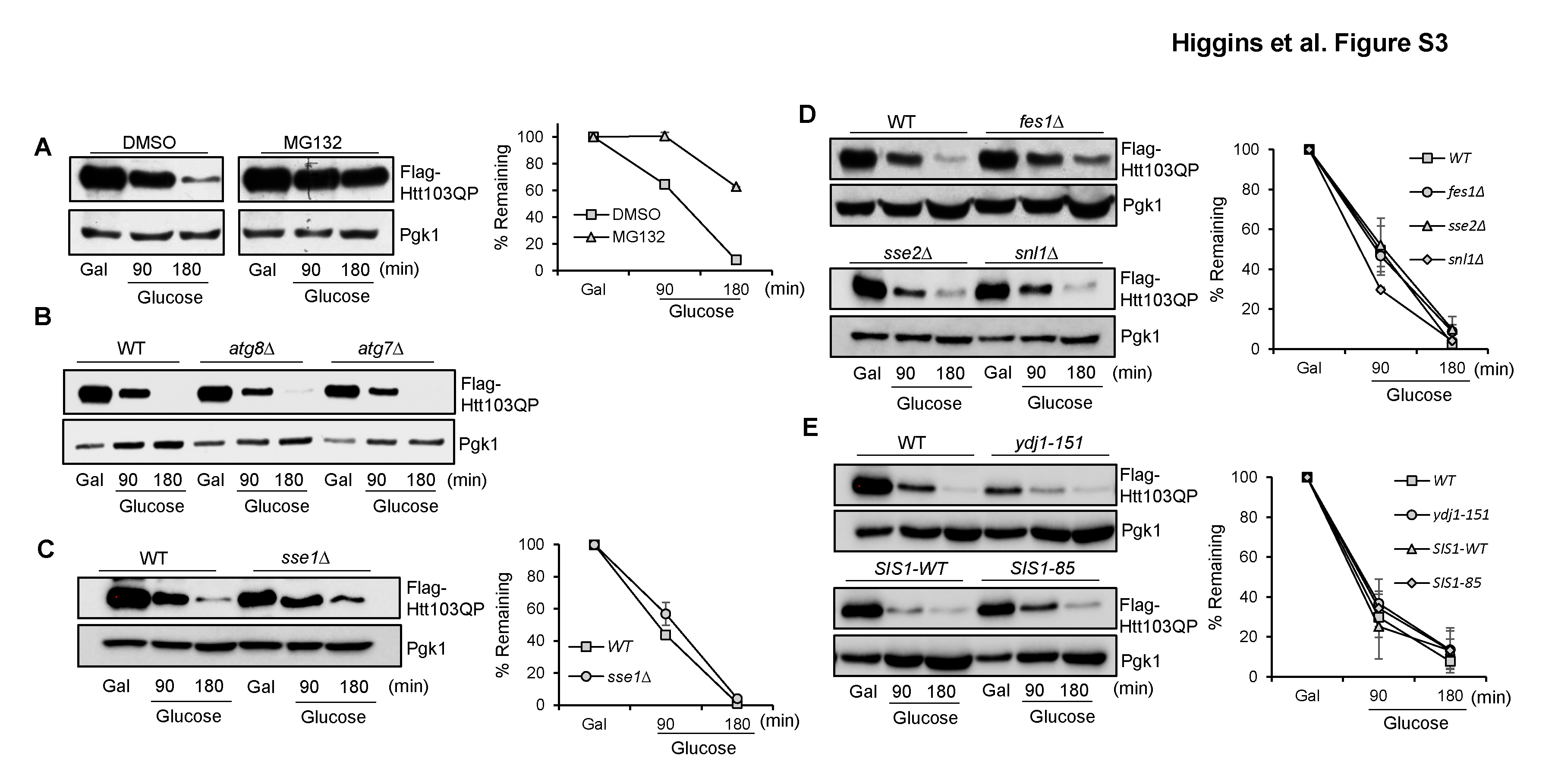

Supplement: S3 Fig — (A) Proteasome inhibitor MG132 blocks Htt103QP degradation after short-time induction. WT cells growing at 30°C in non-inducible (YEP + raffinose) medium containing 0.1% L-proline were treated with 0.003% SDS for 3 hr. Then, either DMSO or 75μM MG132 was added to the cultures for 30 min. Galactose was then added to induce Htt103QP overexpression for 1 hr. Finally, glucose was added to shut off Htt103QP expression. Cells were collected when in galactose (Gal) and also at 90 and 180 min after glucose addition. The Htt103QP protein levels were detected using anti-Flag antibody. Pgk1: loading control. The quantitative degradation kinetics expressed as percent remaining is shown in the right panel. (B) Htt103QP degradation in cells lacking autophagy genes after short-time induction. WT, atg8Δ, and atg7Δ were grown in non-inducible YEP + raffinose medium to mid log phase, then galactose was added for 1 hr to induce Htt103QP overexpression. Glucose was then added to shut off Htt103QP expression. Samples were collected after 1 hr galactose induction and after glucose addition for 90 and 180 min. The Htt103QP protein levels were detected using anti-Flag antibody. Pgk1: loading control. (C) Htt103QP degradation in WT and sse1Δ mutant. Same protocol was used as in (B). The quantitative degradation kinetics expressed at percent remaining is shown in the right panel. (D) Htt103QP degradation in WT, sse2Δ, snl1Δ and fes1Δ cells. Same protocol was used as described in (B). The quantitative degradation kinetics expressed at percent remaining is shown in the right panel. (E) Htt103QP degradation in WT and ydj1-151 cells. Cells were grown at 25°C in non-inducible YEP + raffinose medium to mid log phase, then galactose was added to induce Htt103QP overexpression for 50 min. Cells were then shifted to 37°C for 10 min before glucose was added. Samples were taken after 1 hr galactose induction (Gal) and after glucose addition for 90 and 180 min. The quantitative Htt103QP degradation is s [file pone.0191490.s004.tiff]

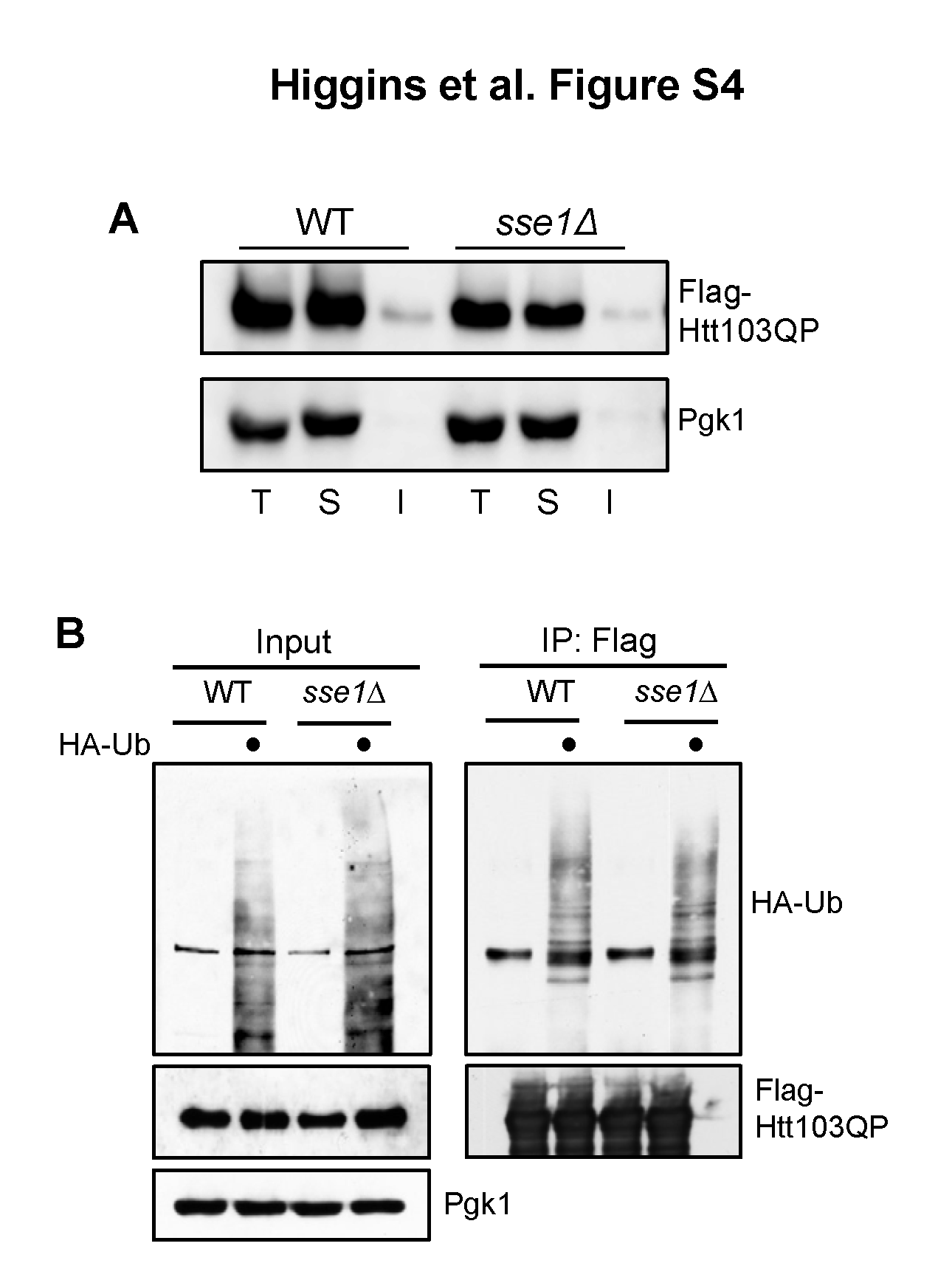

Supplement: S4 Fig — (A) Htt103QP sedimentation assay in WT and sse1Δ cells. Cells were grown in non-inducible raffinose medium at 30°C to mid-log phase, then galactose was added to 2% for 2 hr. Cells were lysed with beads beater and Htt103QP was fractionated into soluble (S) and insoluble (I) fractions by centrifugation. The preparation of the T (total), S and I fractions was described in the Materials and Methods section. Anti-Flag antibody was used to detect Htt103QP, and anti-Pgk1 antibody was used to determine the distribution of Pgk1 in each fraction. (B) Htt103QP ubiquitination in WT and sse1Δ cells. WT and sse1Δ cells carrying PGALFlag-Htt103QP-GFP and PGALHA-Ub or PGAL-HA were grown in raffinose containing medium to early log phase. Galactose was added to induce Htt103QP and Ub overexpression for 4 hr. Flag-Htt103QP-GFP was immunoprecipitated (IP) using anti-Flag M2 agarose beads. Anti-Flag antibody was used to detect Htt103QP protein level. Anti-HA antibody was used to detect Ub protein level. Pgk1: loading control. (TIFF) [file pone.0191490.s005.tiff]
